# Exploring the sorghum race level diversity utilizing 272 sorghum accessions genomic resources

**DOI:** 10.3389/fpls.2023.1143512

**Published:** 2023-03-17

**Authors:** Pradeep Ruperao, Prasad Gandham, Damaris A. Odeny, Sean Mayes, Sivasubramani Selvanayagam, Nepolean Thirunavukkarasu, Roma R. Das, Manasa Srikanda, Harish Gandhi, Ephrem Habyarimana, Eric Manyasa, Baloua Nebie, Santosh P. Deshpande, Abhishek Rathore

**Affiliations:** ^1^ Center of Excellence in Genomics and Systems Biology, International Crops Research Institute for the Semi-Arid Tropics (ICRISAT), Hyderabad, India; ^2^ School of Plant, Environmental and Soil Sciences, Louisiana State University Agricultural Center, LA, United States; ^3^ Wageningen University and Research, Wageningen, Netherlands; ^4^ Genomics and Molecular Breeding Lab, Indian Council of Agricultural Research (ICAR) - Indian Institute of Millets Research, Hyderabad, India; ^5^ International Crops Research Institute for the Semi-Arid Tropics, Hyderabad, India; ^6^ Department of Statistics, Osmania University, Hyderabad, India; ^7^ International Maize and Wheat Improvement Center (CIMMYT), Nairobi, Kenya; ^8^ Sorghum Breeding Program, International Crops Research Institute for the Semi-Arid Tropics, Nairobi, Kenya; ^9^ International Maize and Wheat Improvement Center (CIMMYT), Dakar, Senegal; ^10^ Hytech Seed India Private Limited, Hyderabad, India; ^11^ Excellence in Breeding, International Maize and Wheat Improvement Center (CIMMYT), Hyderabad, India

**Keywords:** sorghum race, deep learning, deep variant calling, *k-mer* analysis, selection pressure, gene enrichment, positive and negative selection

## Abstract

Due to evolutionary divergence, sorghum race populations exhibit significant genetic and morphological variation. A *k-mer*-based sorghum race sequence comparison identified the conserved *k-mer*s of all 272 accessions from sorghum and the race-specific genetic signatures identified the gene variability in 10,321 genes (PAVs). To understand sorghum race structure, diversity and domestication, a deep learning-based variant calling approach was employed in a set of genotypic data derived from a diverse panel of 272 sorghum accessions. The data resulted in 1.7 million high-quality genome-wide SNPs and identified selective signature (both positive and negative) regions through a genome-wide scan with different (iHS and XP-EHH) statistical methods. We discovered 2,370 genes associated with selection signatures including 179 selective sweep regions distributed over 10 chromosomes. Co-localization of these regions undergoing selective pressure with previously reported QTLs and genes revealed that the signatures of selection could be related to the domestication of important agronomic traits such as biomass and plant height. The developed *k-mer* signatures will be useful in the future to identify the sorghum race and for trait and SNP markers for assisting in plant breeding programs.

## Introduction

The process of domestication and natural selection leads to an increased frequency of favorable alleles and subsequently results in complete fixation at target genomic loci (Smýkal et al., 2018). Although the selection process targets advantageous alleles, it also inadvertently results in an increase in the frequency of alleles at neutral loci that are in linkage disequilibrium, a phenomenon referred to as selective sweep (Stephan et al., 1992). A selective sweep has the potential of enhancing the fitness of an individual at the expense of the overall genetic diversity of a population at the respective loci. As a result, modern cultivars are derived from a small fraction of genetically related varieties (Mccouch et al., 2013) in spite of the existence of the vast genetic diversity of global plant germplasm. A better understanding of and stepwise exploitation of existing natural variation in each crop is one key aspect of meeting the increasing food demand in the coming decades.

Sorghum [*Sorghum bicolor* (L.) Moench] is an important cereal crop grown and consumed by a large proportion of the global population. The earliest record of sorghum seeds was recorded at Nabta Playa (Egyptian-Sudanese border) and indicated early domestication (Wendorf et al., 1992). The subsequent migration and adaptation of sorghum across Africa and Asia led to the evolution of morphological and geographically diverse groups, classified into major races (Harlan and Wet, 1972; Harlan and Stemler, 2012). More recent phenotype and genotype-based classifications also support the sorghum race classification within the global diversity panel (Brown et al., 2011). However, inter-racial diversity has not been fully understood in sorghum in a way that allows exploitation of racial structure for heterotic gains. Development of such knowledge would improve overall genomic predictions in sorghum as has been done in other cereal crops (Norman et al., 2018) for the best use of the genome in crop improvement programs.

The extent of genetic diversity is measured by the number of nucleotide variants across individuals and species (Deu et al., 2006; Kebbede, 2020). Such variants range from single nucleotides to large-scale structural differences. However, most studies in the past have only used single nucleotide variation (Afolayan et al., 2019; Enyew et al., 2022) ignoring other structural variations such as insertion-deletions (indels) and presence-absence variations (PAV) (Saxena et al., 2014). PAVs are present in some individuals but absent in others, making them perfect for detecting major differences among multiple genomes. Pangenomes, therefore, can help obtain a more complete set of genomic variants for a species (Hurgobin and Edwards, 2017) since they represent irreversible changes for a given species. The availability of sorghum pangenomes (Ruperao et al., 2021; Tao et al., 2021) makes it possible to carry out a more extensive genetic variation analysis across the different races.

Despite emerging advances in sequencing technologies, distinguishing accurate genetic variants from sequencing errors remains challenging. Because a majority of the genome assembly tools are based on the *de Bruijn* graphs (Zerbino and Birney, 2008; Simpson et al., 2009; Bankevich et al., 2012; Peng et al., 2012), in which the sub-sequence of *k-mer*s (substrings of length *k*) are used to construct the graph and output the paths as contigs (without branching). The resulting contigs can therefore be biased and fragmented as a result of sequencing errors, especially in highly repetitive genomes, leading to low confidence in variant calling. Alternative alignment-free methods of variant detection have been developed using both *k-mer* frequencies and information theory (Song et al., 2014; Pajuste et al., 2017; Zielezinski et al., 2017; Audano et al., 2018). These alignment-free methods have been applied in several studies including for phylogeny estimation (Haubold, 2014), identification of mutations between strains (Nordström et al., 2013) and association mapping (Sheppard et al., 2013).

More recently, deep learning methods have been introduced as a machine learning technique applicable to a range of fields including genomics. Deep learning models can be trained without prior knowledge of genomics and next-generation sequencing (NGS) data to accurately call genetic variants (Telenti et al., 2018). Learning a deep convolutional neural network-based statistical relationship between aligned reads, a genotype calling approach has been implemented in DeepVariant programs (Poplin et al., 2018). The DeepVariant approach is reported to outperform the existing variant calling tools (Poplin et al., 2018).

The objective of our study was to use deep learning (DeepVariant method) to better understand genetic variation, domestication events and selection signatures across known sorghum races. We used existing whole-genome sequence data to quantify genome-wide positive and negative selected regions to enhance our understanding of genome function and the frequency of genetic variations. In addition, we determined the putative signals of selection in sorghum that have resulted from true selective events or population bottlenecks.

## Results

### DeepVariant calling and annotation

**Figure 1:**
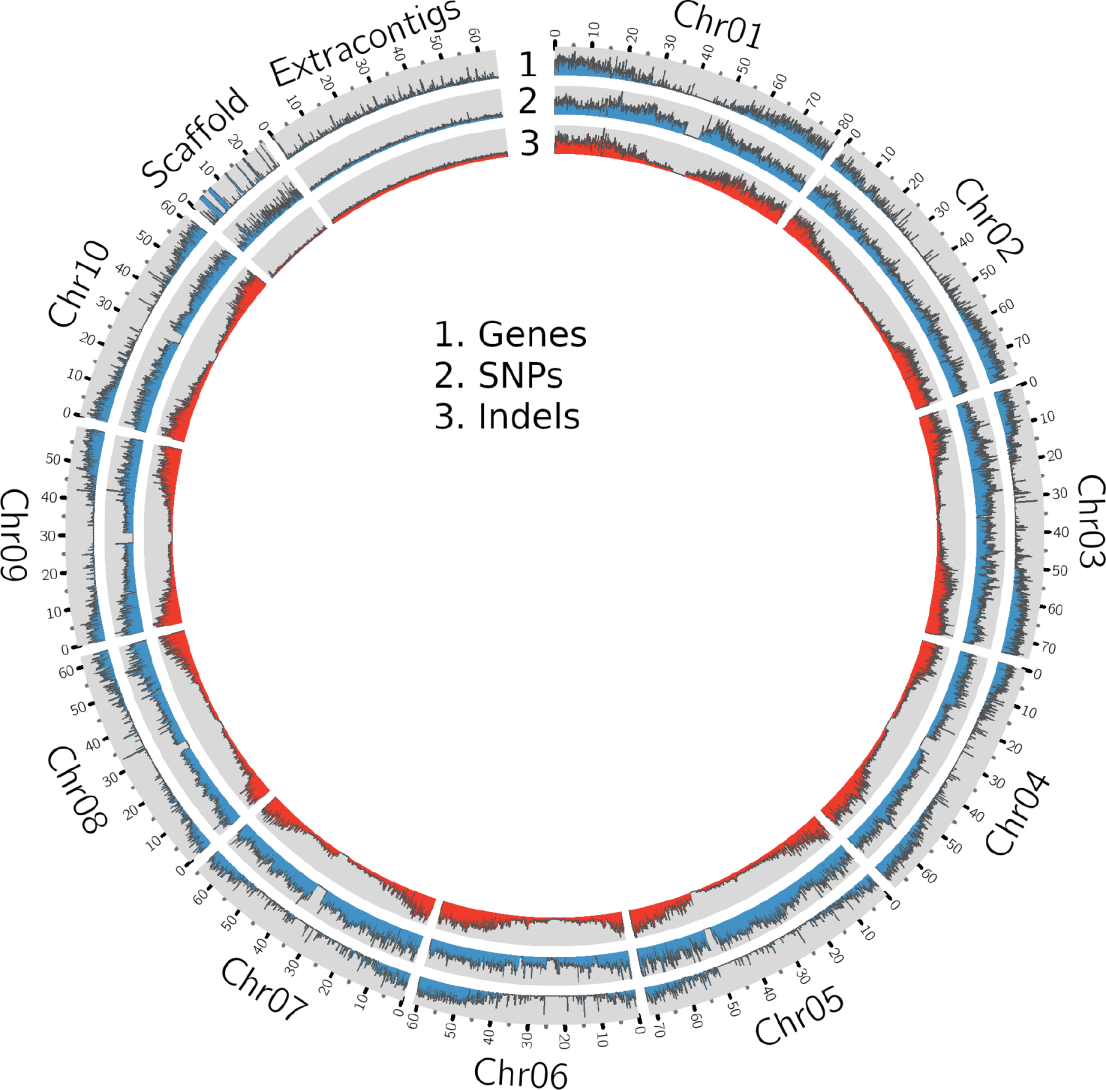
A Circos plot showing the density of genes, SNPs and InDels in the sorghum pangenome.

The whole genome sequence (WGS) data (**Table 1** and **Supplementary Table 1**) were mapped (**Supplementary Table 2**, **Supplementary Figure 1**) to the sorghum pangenome (Ruperao et al., 2021), and a total of 1.7 million high-quality SNPs, and 470,375 InDels (154,900 insertions, 278,951 deletions and 36,524 mixed variants) were called using the DeepVariant method. Homozygous SNPs were predominant (88.3%) over heterozygous SNPs (11.6%) (**Supplementary Table 3**). The overall density of SNPs was 2.5 SNP/kb, whereas the indel density was 0.6/kb. The maximum (209,429) and minimum (147,952) SNPs were reported on chromosome 2 (0.3/kbp) and chromosome 9 (0.4/kbp), respectively (**Supplementary Table 4**) (**Figure 1**), while the maximum (70,722) and minimum (34,530) number of indels were reported on chromosome 1 (0.8%) and chromosome 8 (0.5%) respectively. Most of the insertions (98%) and deletions (93%) were less than 10bp in length (**Supplementary Figure 2A**).

**Table 1:** A summary of publicly available data used in the current analysis.

Reference	# Genotypes	Average coverage
Valluru et al. (2019)	196	13x
Jensen et al. (2020)	70	7x
Yan et al. (2018)	6	28x
**Total**	**272**	**16x**

SNP annotation reported 11% SNPs of which 51,891 were synonymous and 53,159 non-synonymous, resulting in a non-synonymous-to-synonymous substitution ka/ks ratio of 1.02 (**Supplementary Tables 5**, **6**), consistent with the previous study by Mace et al. (2013). Sorghum accessions NSL54318 (50,238 non-synonymous; 3,933 start gain; 88 start lost and 611 stop gain SNPs) and PI660645 (46520 non-synonymous; 3700 start gained and 103 stop lost) harbored the maximum and minimum effect SNPs (**Supplementary Table 5**). There were more transitions (C/T and A/G) than transversions (A/T, A/C, T/G and C/G) with a transition/transversion ratio ranging from 1.912 (NSL50716, IS30508) to 1.983 (PI329719). The overall tr/tv ratio was 1.960 (**Supplementary Table 7**).

SNPs with large effects were the least common (1,362; 0.04%) compared to SNPs with low (63,298; 1.9%), moderate (53,159; 1.6%) and modifying SNPs (96%). A total of 89.3% (1,595,340) of the SNPs were conserved across five sorghum race accessions while the remaining 10.6% (190,321) were variably detected in at least one sorghum race. Among the SNPs in the sorghum race accessions, 0.03% (590) were race-specific, the majority (60.6%; 358) of which were reported in durra and the least in the bicolor race (6.1%; 36) (**Supplementary Table 8**) (**Supplementary Figure 2B**). Most of the race-specific SNPs (57.9%) were highly confident with support from more than 10 accessions. Only 21% of the race-specific SNPs were supported by less than 5 accessions (**Supplementary Figure 2C**).

Sorghum races caudatum, durra, guinea and kafir had the highest proportion of SNPs with the low MAF category (0.0,0.1) compared to bicolor. Kafir had the highest proportion of SNPs with MAF category (0.1, 0.2) while the bicolor race reported the highest proportion of SNPs with MAF greater than 0.2, which is expected for a race with a long history of cultivation (**Supplementary Figure 2D**).

### Genetic and nucleotide diversity

The SNP-based Neighbor-Joining (NJ) dendrogram of the 272 genotypes grouped them largely according to race genetic relatedness (**Supplementary Figure 3**). Four major clusters were observed with a number of subgroups. The phylogenetic tree contained a distinct cluster of 63 guinea race accessions (nodes in blue color) mixed with a few other race individuals, such as durra (PI221662, PI248317, PI267653 and PI148084) (nodes in brown color), kafir (PI660555 and NSL365694) (nodes in pink color), bicolor race (IS12697) (nodes in red color). The other sorghum race clusters were split with non-corresponding sorghum race accessions. For example, durra has 91 accessions split into two clusters with caudatum and kafir accessions. The bicolor accessions were placed mostly in durra and guinea clusters. Among the bicolor accessions, the China origin accessions were grouped distinctly in the durra cluster compared to other bicolor accessions.

The evaluation of nucleotide diversity across all 272 accessions showed that sorghum had low diversity (0.0000483715) compared to wheat (*π_A_* =0.0017, *π_B_* = 0.0025 and *π_D_* = 0.0002) (Zhou et al., 2020), maize (*π* = 0.014) (Tenaillon et al., 2001) and rice *π* = 0.0024 (Huang et al., 2010) (**Supplementary Figures 4, 5**). The diversity varies depending on the population size and the level of diversity of the accessions used in such a population. However, such low diversity was also reported in an earlier study (Sapkota et al., 2020). We observed significant differences (*P* < 0.05) in nucleotide diversity between three sorghum races (caudatum, durra and guinea) that were represented with more than 50 genotypes. The durra had the highest nucleotide diversity while caudatum showed the lowest (*π_C_* = 0.0000419, *π_G_* = 0.0000631 and *π_D_* = 0.0000637). The distribution of nucleotide diversity on the sorghum race genome was in the order of *π_D_* > *π_G_* > *π_C_*.

We used the *Fst* index to estimate the temporal genetic divergence between the race accessions and observed that the level of genetic differentiation among the sorghum race populations ranged from moderate (*Fst* = 0.044 for caudatum vs durra) to relatively high (*Fst* = 0.18 for bicolor vs guinea) (**Supplementary Tables 9, 10**; **Supplementary Figure 6**) indicating that inter-population differences were relatively low. The average *Fst* between the bicolor and other races was ~0.16, which was higher than in non-bicolor race comparisons suggesting that gene flow from bicolor to other races was much earlier than the gene flow between the rest (non-bicolor) of the races. The durra and guinea populations revealed the second-highest *Fst* of 0.1228 and were classified as the sorghum race intermediates (**Supplementary Table 9**). A total of 19,696 SNPs having significant high *Fst* were reported between bicolor*-*kafir race combinations, of which 910 SNPs were genic SNPs (**Supplementary Table 10**).

The difference between (diverse) sorghum race populations was measured with Tajima’s D (**Table 2**). A total of 13,070 SNPs were reported to have *θ_π_* (observed value) less than *θ_k_* (expected value) (maximum 4,612 and minimum 1,869 SNPs from durra and bicolor respectively), indicating that the variants may have undergone a recent selective sweep. Another 311,045 SNPs reported greater *θ_π_* compared to *θ_k_* (maximum 202,684 and minimum 76,836 SNPs from guinea and bicolor, respectively) suggesting balancing selection. Compared to non-bicolor race mutations, a lower number of mutations were linked to genes within a selection sweep than with balancing selection genes in the bicolor race (**Supplementary Tables 11**, **12**).

**Table 2:** Summary SNP statistics in Sorghum race populations.

Race	SNP	*π* (10^-5^)	Tajima’s D (*θπ*>*θk*)	Non-synonymous SNPs	Synonymous SNPs	Non-synonymous/Synonymous
Bicolor	374,545	3.37	0.44	11,100	9,957	1.114
Caudatum	961,644	2.72	0.27	31,391	31,168	1.007
Guinea	976,558	4.30	0.37	31,827	31,681	1.004
Durra	1,261,078	3.89	0.18	38,362	37,651	1.018
Kafir	886,880	4.72	0.26	27,779	27,458	1.011

### *K-mer* based divergence

**Figure 2:**
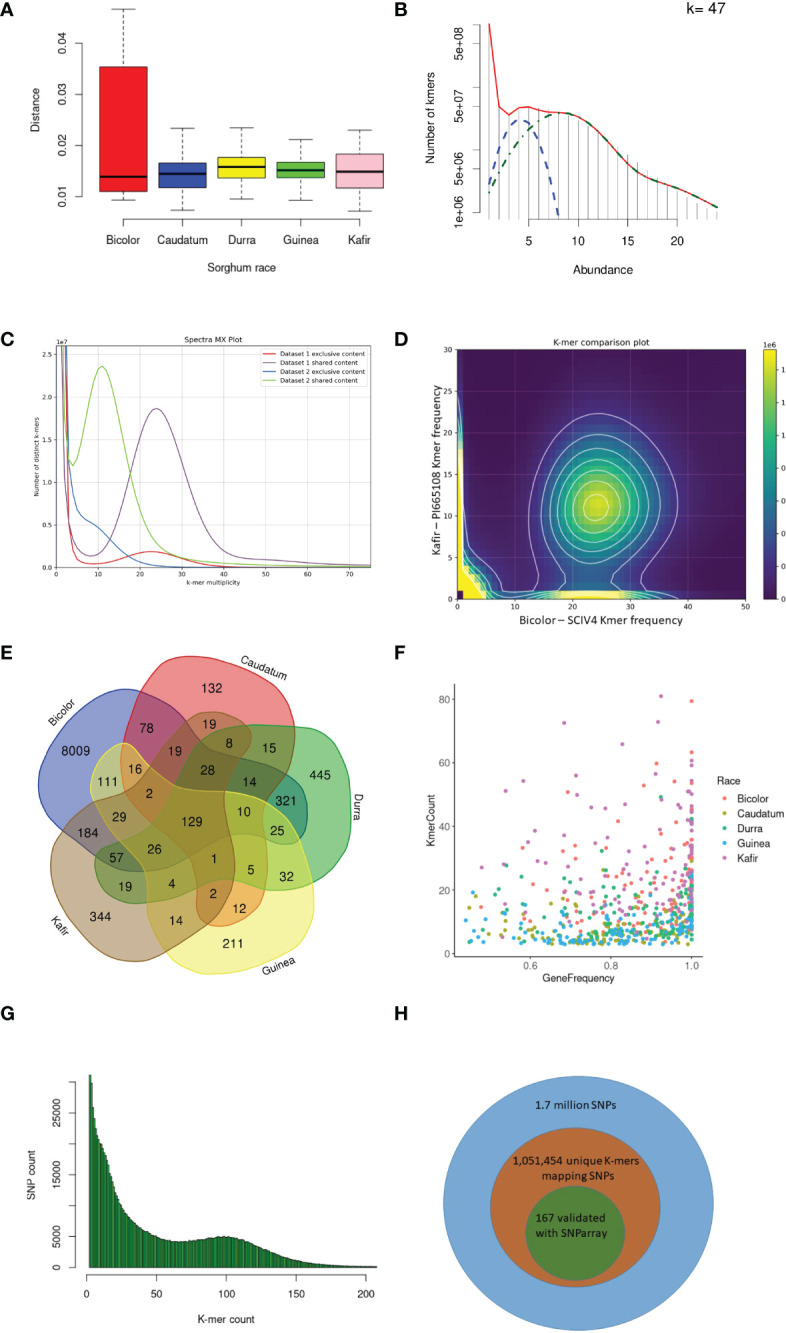
*K-mer* and read mapping overview. **(A)** An alignment-free method, the Jaccard index uses the hash procedure to measure the distance between sorghum race accessions. **(B)** Sampled histogram and fit for 47 *k-mer* lengths. Red is the fit of the complete statistical model of the histogram, blue is the heterozygous *k-mers* and green is the only homozygous *k-mers* **(C)** The *k-mers* share between kafir (PI665108) and bicolor (SCIV4) sorghum race accessions as dataset1 and dataset2 respectively. **(D)** Distinct *k-mer* share between kafir and sorghum race, the cloud indicates the shared *k-mers* and heigh density *k-mers* on x and y-axis are unique *k-mers* respectively. **(E)** Mapped *k-mer* sequence reads in the number of genes in each sorghum race accessions **(F)** Proportion of sorghum race common genes covered with horizontal and vertical coverage with *k-mer* sequence reads. **(G)** The unique *k-mer*s holding the deepvariant SNPs with respective *k-mer* count and off these, **(H)** the proportion of deepvariant SNPs validated.

The *k-mer* genetic distance between the sorghum accessions was computed from the size-reduced sketches and distance function developed in the mash tool (**Supplementary Table 13**). The durra race was the most distinct from the reference pan-genome (Ruperao et al., 2021) based on the mean distance of accessions, followed by guinea (**Figure 2A**). The bicolor race was the most closely related race to the reference (**Figure 2**). Accessions from each sorghum race, SCIV4, PI285039, PI276823, PI665088 and PI665108 from bicolor, caudatum, durra, guinea and kafir, respectively were more genetically distinct from the reference (**Supplementary Table 13**) and representative of the specific race and therefore used for *k-mer* analysis. These distinct sorghum accessions were in agreement with the NJ distance between the accessions (**Supplementary Figure 3**).

With the optimized 47 *k-mer* size (**Figure 2B**), the overall *k-mer* sequence comparison between the five race accessions (2.3 billion *k-mer*s) showed that 35.3% (434 million unique *k-mer*s) of common *k-mer*s present in all five races accessions, this indicates the conserved *k-mer* of all sorghum race accessions. The 13.3% (314 million *k-mers*) were commonly seen in any four sorghum race accessions, indicating that these *k-mers* were absent in at least any one of the sorghum races. This variability decreased to 8.8% (108 million *k-mers*) and 6.3% (78 million *k-mers*) on measuring the common *k-mers* between three and two sorghum race accessions respectively. For example, SCIV4 (bicolor) and PI665108 (kafir) shared 402 million distinct *k-mers*, which was 45% and 23.5% of total distinct *k-mers* reported respectively (**Figures 2C-D**). From this *k-mer* comparison between the sorghum race accessions, 23.8% of *k-mer*s were unique to sorghum races. These race-specific *k-mer*s were possibly unique to genomic sequence (as a single genome sequence for each race was used for the analysis).

Overall, 10,321 gene PAVs were identified based on the *k-mer* sequence reads mapping to sorghum pan-genome assembly (**Supplementary Table 14**) (**Figure 2E**). The mapping of the race-specific *k-mer* sequence reads identified 132, 8009, 211, 445, and 344 unique genes in caudatum, bicolor, guinea, durra, and kafir sorghum accessions, respectively. One hundred and twenty-nine (129) genes were commonly present in all sorghum race accessions (**Supplementary Table 15**), indicating the *k-mer*s are unique with the specific variations or *k-mers* partially mapping the gene length-frequency with horizontal mapping range of 0.4 to 1 (frequency) (**Figure 2F**). Furthermore, 1,051,453 SNP were identified supporting the *k-mer*s sequence (**Figure 2G**) reads of which, 85,048 SNPs were genic, and 167 SNPs were validated with the SNParray sequences (**Figure 2H**) (**Supplementary Table 16**) used for sorghum pangenome analysis (Ruperao et al., 2021).

### Selection signatures

**Figure 3:**
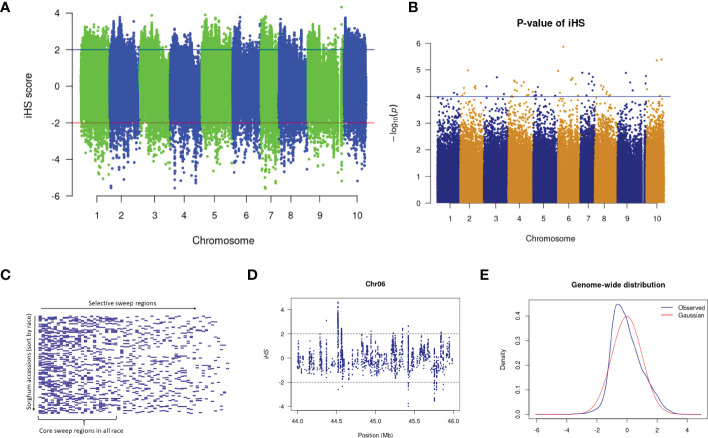
**(A)** The iHS score distribution across the genome with **(B)** associated p-values. **(C)** Selective sweep regions in sorghum accessions sorted by race. **(D)** A subset of iHS on chromosome 6 showing the regions having values above and below the threshold value and **(E)** The distributions of the standardized iHS scores and comparison with standard Gaussian distribution.

Several sweep regions were detected with iHS (**Figures 3A, B** and **Supplementary Figure 3**), of which, 64 were significant (FDR < 0.05) (**Supplementary Table 17**). The majority of sweeps were reported on chromosome 7 (19 regions) followed by chromosome 4 (17 regions) and chromosome 10 (2 regions) (**Supplementary Table 17**). The highest number of selective sweep regions were observed in durra (54 regions), followed by caudatum (51), guinea (45), kafir (38) and bicolor (30) (**Supplementary Tables 18**, **19**). A total of 14 selective sweep regions were common in all five sorghum races while 21 regions were uniquely absent in any one sorghum race (**Supplementary Table 18**). For example, 9 selective sweep regions were reported in four sorghum races but uniquely absent in the bicolor race alone (**Supplementary Table 18**).

We used the cross-population extended haplotype homozygosity (XP-EHH) score and detected sweep regions from each combination of sorghum race population (**Supplementary Figure 7**) (**Table 3**). We identified 8,888 significant (FDR < 0.05) selection sweep regions, of which 3,504 regions were common between more than two sorghum race combinations (**Supplementary Tables 20**-**S29**). Out of all selective sweeps identified from the sorghum race combinations, chromosome 5 had the maximum of 1,399 regions while chromosome 9 had the least (616). The kafir population exhibited the highest (2,473) selective sweep regions in comparison with the guinea race (**Supplementary Table 28**). Only a few (525) sweep regions were reported in bicolor population (**Supplementary Table 23**).

**Table 3:** Description of the candidate selective sweep regions detected using XP-EHH between the sorghum race populations.

Sorghum race combination	Significant XP-EHH regions	Supporting iHS	XP-EHH candidate genes$	XP-EHH mapping genes&
Bicolor × Caudatum	671	3	212	109
Bicolor × Durra	640	3	183	127
Bicolor × Guinea	612	5	201	127
Bicolor × Kafir	522	0	117	61
Durra × Caudatum	1,832	1	587	414
Durra × Guinea	2,350	4	677	367
Durra × Kafir	1,761	3	424	356
Guinea × Caudatum	2,084	6	607	328
Guinea × Kafir	2,070	1	711	426
Kafir × Caudatum	1,263	2	425	365

$ The number of genes refers to the genes mapping 5kb upstream/downstream to selective sweep region. & The selective sweep region present within the gene regions.

### Overlapping selection regions between Tajima’s D and XP-EHH

**Figure 4:**
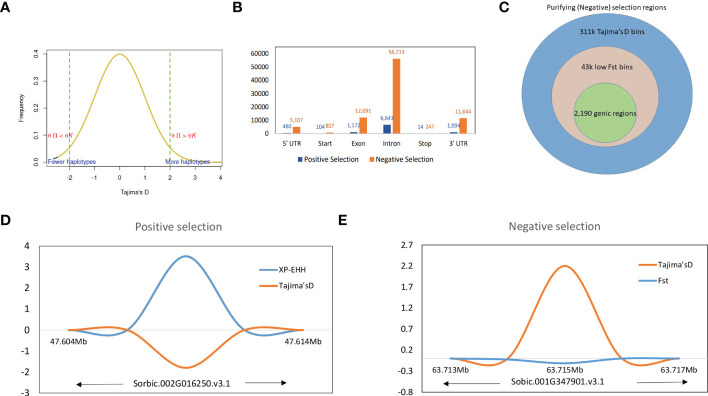
**(A)** Tajima’s D values distribution with signifying positive and negative thresholds. **(B)** Structural annotations of genomic regions in positive and negative thresholds. **(C)** The proportion of higher Tajima’s D values and lower *Fst* valued bins as negative selection regions and corresponding genic regions. **(D)** The positive selection region on Chr09 with XP-EHH score and Tajima’s D valued plot and **(E)** A genic negative selection region on Chr01 (specific to guinea race) with significantly higher Tajima’s D and lower *Fst* value regions.

We defined the overlapping selection regions as those located beyond the thresholds and in the same chromosome sequence location. Tajima’s D statistics were obtained from each sorghum race population dataset and identified the genes which did not fit the neutral theory model at equilibrium between mutation and genetic drift. A total of 324,115 genome-wide bins were observed with non-equilibrium statistics of neutrality test, of which 311,045 (with SNPs in range of 76,836-bicolor to 202,684-guinea) were undergoing purifying selection (negative selection) and 13,070 were (with SNPs in range of 1,869-bicolor to 4,612 durra) selection maintained (balanced positive selection) (**Figure 4A**). Among the variants undergoing purifying selection, 43,191 bins had a significant low *Fst* index supporting the signature of a recent population expansion (**Figure 4B**), of which 14% were from genic regions (**Supplementary Table 30**). The purifying selection regions had low diversity (**Figure 4D**) with reduced allele frequency in the descendant population compared to the ancestral population.

The significant selection regions (FDR <0.05) detected by XP-EHH were specific to the pair-wise sorghum race combinations. Among the identified 8,888 significant XP-EHH candidate-sweep regions from overall sorghum race combinations, of which 179 regions were genic (**Supplementary Table 31**) and “selection-maintained variations” indicating the recent population contraction. The overall sweep regions were in the comparable range identified in other crops such as wheat, (3,105 – 16,141 sweep regions in the genome of domesticated einkorn and emmer lines; Zhou et al., 2020) and soybean (3,811 genes positioned within the selective sweep regions)(Saleem et al., 2021). Among the chromosomes, chromosome 5 and groups of 4,7,9,10 chromosomes contained the highest (33) and lowest (14) numbers of genes, respectively. A relatively low Tajima’s D was observed in selective sweep regions when compared with a significantly higher XP-EHH valued region (**Figure 4D**).

### Enrichment of candidate genes under selection

A total of 2,370 genes genome-wide were observed to deviate significantly using equilibrium/neutrality tests, of which 179 were selection-maintained (balanced selection) while 2,191 were undergoing purifying selection (**Supplementary Tables 30**, **31**). Durra and bicolor had the maximum (110) and minimum (39) number of genes undergoing positive selection respectively. Bicolor (409) and guinea (1,133) had the maximum and a minimum number of selection-maintained genes.

**Figure 5:**
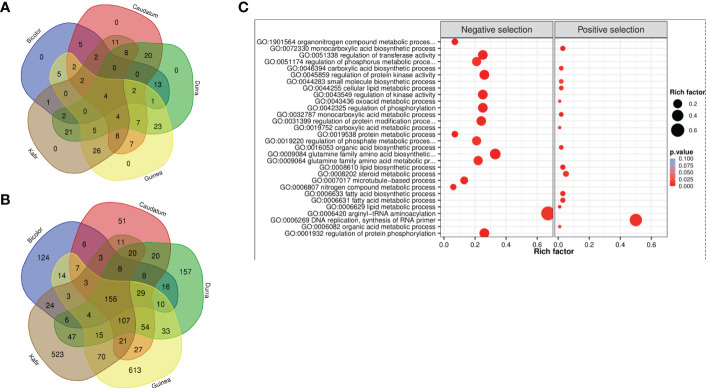
Venn diagram of **(A)** positive selection and **(B)** negative selection genes from five sorghum races combinations. **(C)** Significantly enriched GO biological terms for positive and negative selected genes (bubble color indicates the p-value range and size indicates the gene ratio).

A similar trend of the fewest number of genes were reported in the bicolor race (421), with guinea having the maximum (1,166) genes under purifying selection. Among the five races, guinea and kafir shares the maximum number of common selection-maintained (26) and purifying (70) genes, suggesting potential rich gene flow between these two races (**Figure 5**). Additionally, guinea, kafir and durra reported maximum genes as sweep regions (guinea-kafir: 26, durra-guinea: 23 and durra-kafir: 21) (**Figure 5A**), with low nucleotide diversity (*π*) in caudatum and bicolor (**Figure 3B**) also indicating the traits regulated by these regions may have undergone similar histories of selection.

The 2,370 genes undergoing selection pressure (both positive and negative) showed significantly enriched gene ontology (GO) term and among these genes (**Figure 5C**, **Supplementary Figure 8**), the top GO term was lipid biosynthetic process (GO:0008610) and organonitrogen compound metabolic process (GO:1901564) for genes with positive and negatively selection, respectively (**Supplementary Table 32**). Among the positively selected gene set, most of them were enriched with lipid biosynthetic process (GO:0008610), metabolic process (GO:0006629), carboxylic acid metabolic process (GO:0019752), oxoacid metabolic process (GO:0043436), organic acid metabolic process (GO:0006082), most of these metabolic pathways were related to plant stress resistance. Whereas the negatively selected gene was majorly enriched with nitrogen compound metabolic process (GO:0006807), organonitrogen compound metabolic process (GO:1901564) and protein metabolic process (GO:0019538) (**Supplementary Table 32**). The nitrogen utilization and metabolic pathway were found significantly enriched and confirmed the genes under selection throughout either domestication or during subsequent breeding with earlier selection study (Massel et al., 2016). The genes enriched with ‘DNA replication, ‘lipid metabolism’ and ‘hormone signal’ suggest that sorghum has evolved defense strategies, and enrichment of phosphorylation, kinase activity, transferase, phosphate and phosphorus metabolic process triggers many metabolic processes and plant growth activity.

**Figure 6:**
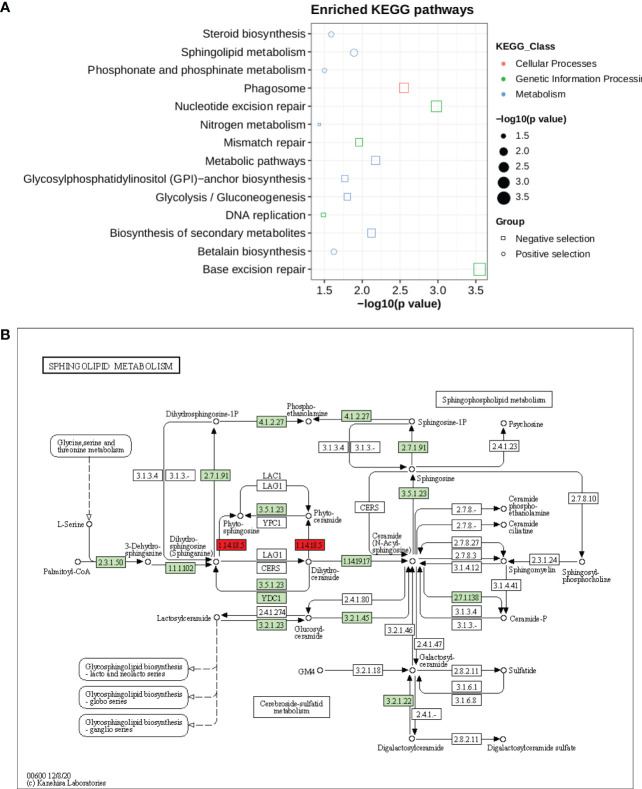
**(A)** KEGG pathway enrichment for genes under selection pressure **(B)** Pathway of sphingolipid metabolism. The two red boxes represent the positively selected genes involved in pathways.

Kyoto Encyclopedia of Genes and Genomes (KEGG) pathways were identified according to the selection’s signature candidate gene with a p-value <0.05 (**Figure 6**). A KEGG pathway enrichment analysis was performed for the selection signature gene to identify the number of significantly changed samples along the pathway that were relevant to the background number. A total of 2,370 genes were mapped onto 315 pathways, and the most enriched sequences were metabolic pathways and biosynthesis pathways. The top 14 pathways with the greatest number of annotated sequences are shown in **Supplementary Table 33**. Most of the significant pathways were in metabolism, biosynthesis, excision repair and secondary metabolites. The most significantly changed KEGG pathways were in sphingolipid metabolism (**Figure 6B**), betalain, steroid biosynthesis, phosphonate and phosphinate pathways for positive selection genes. Sphingolipids are essential components of plasma membrane providing structural integrity to plant membrane, regulating the cellular process, and also enhancing the tolerance of sorghum to biotic and abiotic stresses. Steroid hormone biosynthesis and the phosphonate and phosphinate metabolism pathways are also involved in the adaptation of sorghum to low salinity. Whereas base and nucleotide excision repair, biosynthesis of secondary metabolites, glycolysis, GPI and nitrogen metabolism were significantly enriched in negative selection genes (**Supplementary Table 33**). These annotations provide valuable information for studying the specific biological and metabolic processes and functions of genes under selection pressure in sorghum accessions.

### Overlap of signatures of selection with QTLs

Quantitative trait loci associated with seven traits that overlapped with detected signatures of selection were compared with earlier reported sorghum QTLs (Hostetler et al., 2021). Analysis of the overlaps between signatures of selection and reported QTL indicated that 10 and 206 linked genes were identified as positively and negatively selected genes respectively (**Supplementary Tables 34**, **35**). Some QTL for traits of plant height, root biomass, dead above-ground biomass, live above-ground biomass and total biomass overlapped significantly with putative gene regions of signatures of selection.

## Discussion

We have demonstrated the utility of vast the sorghum genomic data that exists in public databases for characterization of a representative set of sorghum (Valluru et al. 2019). Our results validate the application of deep learning for the characterization of sorghum races and goes further to establish nucleotide diversity and genetic divergence across and within different sorghum races. We also used existing QTL data to identify candidate genes that are under both negative and positive selection.

The sorghum reference set used in the current study was earlier selected by Billot et al. (2013) after genotyping 3,367 global collections using 41 representative nuclear SSR markers and is considered to be representative of the sorghum collections that exist in various global gene banks. Our results confirm that the clustering of sorghum germplasm was largely according to regions (Paterson et al., 2009; Bekele et al., 2013; Ramu et al., 2013) indicating continuous gene flow between various racial groups depending on where the sorghum races are grown. The only distinct exception was in the guinea race, which was expected since the guinea race is specifically grown in West Africa and therefore any gene flow would be confined within the West African locations.

Our study also identified many intermediate accessions (more than 15 accessions) as a result of the continuous gene flow suggesting that a different criterion other than morphology will need to be used in future studies for the correct classification of sorghum races and their intermediates.

Capturing race-specific sequences will be critical in future studies for the follow-up identification of variants and/or, genes associated with each sorghum race. For example, longer k-mers (>15 bp) have been utilized as biomarkers (Drouin et al., 2016; Wang et al., 2018) as they can hold biological information and depict specific signatures in nucleotide sequences (Wang et al., 2016). Our ability to differentiate abundant k-mers between the different sorghum races in the current study provides an opportunity for future studies to utilize k-mers as race- or accession-specific identifiers in sorghum. Currently we were able to identify the sorghum race-specific k-mers, that are present in respective race, and able to locate the position and associate the genomic features. Based on the sequence read mapping, the gene PAV was earlier identified in the sorghum pangenome (Ruperao et al., 2021), and adapting the similar approach the genes having the race specific k-mers regions were also reported. With the known unique k-mer position, it is possible to extend the study of the genomic features having race-specific unique sequence (such as any genetic variations including the SSR, SNP, CNV and SV).

### DeepVariant calling and its utility in sorghum breeding

For the first time in sorghum, we used the DeepVariant (Poplin et al., 2018) tool, a deep learning approach for SNP calling, and reported over two million genome-wide variants from existing sequencing data. One of the concerns of SNP calling from NGS data is the accuracy of SNPs. A recent comparison of SNPs called from the traditional SNP calling tools such as GATK (Depristo et al., 2011) with DeepVariant method reported superior performance of the latter (Lin et al., 2022), further validating our choice to implement this method in sorghum. Our results were largely consistent with previous studies in sorghum that involved SNP calling from NGS data, including the patterns of SNP distribution observed across the genome (Paterson et al., 2009; Bekele et al., 2013) and the non-synonymous to synonymous SNP substitution ratio. In this study, the deepvariant has called the variants with 0.19/Kbp which is comparatively less dense than the earlier reported results (0.33/Kbp) with GATK (Ruperao et al., 2021). Our results were also within the range reported for other genome-wide studies such as in soybean (Lam et al., 2010), rice 1.2 (McNally et al., 2009), and Arabidopsis 0.8 (Clark et al., 2007).

Future studies will need to compare DeepVariant with other existing methods and validate our results in different germplasm sets, such as the sorghum diversity panel (Casa et al., 2008). Such future studies will also need to pay special attention to sequence coverage and how it would affect the accuracy of variants called. Our study used a minimum overall coverage of 5x, which was more than adequate even for a less efficient SNP calling pipeline (Wu et al., 2019). Sequence coverage is one of the major factors affecting the accuracy of SNPs called from NGS datasets, especially in heterozygous species (Gong and Han, 2022). A coverage of 0.01x has been reported as the most cost-effective coverage in sorghum, with 94.1% SNP accuracy (Jensen et al., 2020). There will be a need for additional studies establishing the effect of various levels of coverage in the NGS datasets for DeepVariant calling, and how it would affect the SNP accuracy in sorghum.

### Nucleotide diversity and divergence in sorghum

The genetic relatedness from the NJ tree (**Supplementary Figure 3**) and Pco (**Supplementary Figure 9**) analysis between the sorghum race accession demonstrates most of the guinea accessions forms the cluster, except for few accessions relates to caudatum race. Whereas durra race represented in two clusters, one cluster close to guinea and second in between kafir and caudatum (**Supplementary Figure 3**). Such intermediate race accessions and split of sorghum race clustering was also seen earlier for 389 sorghum diverse panel (Sapkota et al., 2020). On further investigation of structure analysis supports the two subpopulation clusters (K=2) in the sorghum population (**Supplementary Figure 10**) supporting the distance-based NJ analysis, indicating that the race accessions are genetically related. Our study was purely based on existing data and did not allow for much flexibility in the number of genotypes per race. The overall nucleotide diversity observed for sorghum of *π* = 0.000048 is significantly smaller than previously reported by Faye et al. (2019) but comparable to a more recent study (Sapkota et al., 2020) that reported *π* = 0.000032. This figure is much lower than for other cereals such as wheat (*π*_A_=0.0017, *π*_B_ = 0.0025 and *π*_D_ = 0.0002) (Zhou et al., 2020), maize (*π* = 0.014) (Tenaillon et al., 2001) and rice (*π* = 0.0024; Huang et al., 2010) and could be a consequence of the limited number of genotypes used in the study. The race-specific nucleotide diversity indicated that the caudatum (57 genotypes; *π*_C_ = 0.0000419) had the lowest diversity followed by guinea (68 genotypes; 0.0000631) and durra (82 genotypes; *π*_D_ = 0.0000637) races. On comparing the linkage disequilibrium (LD) decay, rapid LD decay was observed in durra followed by guinea and caudatum (**Supplementary Figure 10**), supporting the above diversity values of the sorghum race. The least diversity race population (caudatum) shows the higher extents of LD than the races with higher diversity (Durra). Supporting to these results, caudatum race consistently demonstrate the least genetically diverse showed higher LD values (Sapkota et al., 2020). However, studies also reported, the guinea race as the most genetically diverse sorghum type (Morris et al., 2013; Faye et al., 2019). Comparing our results and those of Morris et al. (2013) and Faye et al. (2019), suggests a positive correlation between the number of genotypes per race with the nucleotide diversity. More studies need to be done to confirm the effective population size per sorghum race that will be optimum for a reliable and consistent nucleotide diversity result.

### Selection signatures

We used two approaches to detect selection sweeps across the sorghum genome, both of which are haplotype-based. The iHS method, which is based on a single population, was meant to detect recent positive selection (Voight et al., 2006), while the XP-EHH is based on the comparison of two populations and is considered powerful in detecting beneficial alleles shortly before, or at fixation (Alexandra et al., 2015). Such multiple statistical approaches were earlier used for selection sweeps in other crops like cotton (*Gossypium herbaceum*) (Nazir et al., 2020) and soybean (*Glycine max*) (Zhong et al., 2022). A recent study comparing different methods used for detecting selection sweeps reported that both iHS and XP-EHH were able to identify genomic regions undergoing selective sweep under a wide range of population structure scenarios (Vatsiou et al., 2016). Previous studies in sorghum have also reported evidence of selective sweeps in sorghum (Casa et al., 2006; Faye et al., 2019) although the methods used for detection were different. Our results on selective sweep regions were further strengthened by Tajima’s D results, which enabled us to identify candidate genes in the significant selective sweep regions.

The 2,370 candidate genes identified in our study (for under selection pressure), of which, 7.5% are positively selected, are similar to the proportion of genes identified for domestication and improvement using the gene-based population study by Mace et al. (2013). The genomic regions that are either positively or negatively selected in the respective sorghum races could give a hint on geographic preferences. More studies will need to delve deeper into specific regional selection sweeps that could eventually be used to predict ideal genotypes/phenotypes. The remaining candidate genes that were reported as undergoing negative selection with evidence from both Tajima’s D and Fst index values. Such genomic analysis of crop landraces would enhance our understanding of the basis of local adaptions (Li et al., 2017; Swarts et al., 2017).

Some of the trait-associated genes undergoing selection pressure that have been reported include the dry pithy stem gene mutation that led to the origin of sweet sorghum (Zhang et al., 2018), local adaptation to parasite pressure and signatures of balancing selection surrounding low germination stimulant (Bellis et al., 2020) and the strong selection pressure on the sorghum maturity gene (Ma3) (Wang et al., 2015). Comparative population genomics assist in dissecting the domestication and genome-wide effects of selection as studied in cotton, with reports that 311 selection sweep regions are associated with domestication and improvement (Nazir et al., 2020) and with selection sweeps identified comparing wild and domesticated soybean accessions (Zhong et al., 2022).

Populations subjected to strong selection pressure may experience genetic bottlenecks and result in a loss of genetic diversity. The level of diversity preserved in a population depends on the background of the emerging adaptive alleles (Wilson et al., 2017). Identification of such a large number of selection sweeps suggests the existence of domestication bottlenecks. The identified selection sweeps overlapped with highly differentiated regions suggesting the occurrence of differentiation due to human-mediated selection. These regions help in understanding the genetic basis of domestication and improvement in traits. On further comparison of the selection regions with significant loci of GWAS analysis (narrowing down the region), it may be possible to determine the genes underlying domestication and selection in the sorghum crop.

The results from this study lead to a better understanding of the changes at the genomic level caused by domestication, selection and improvement of sorghum accessions.

## Methods

### Plant material

We used 272 sorghum accessions, which included accessions that had been used in a previous sorghum pangenome study (Ruperao et al., 2021) and six sorghum bicolor accessions reported in Yan et al. (2018) (**Supplementary Table 1**). Among these genotypes, 82, 21, 57, 68, 14 were durra, kafir, caudatum, guinea and bicolor respectively, while the remaining were mixed accessions.

### Variant discovery

The fastq sequence reads generated from the 272 sorghum accessions were trimmed with Trimmomatic 0.39 (Bolger et al., 2014). Alignments to the sorghum pangenome (dataverse.icrisat.org, https://doi.org/10.21421/D2/RIO2QM) as a reference (Ruperao et al., 2021) were performed using Bowtie2 version 2.4.2 (Langmead and Salzberg, 2012). All alignments were converted to binary files with Samtools 1.13 (Li et al., 2009) followed by filtering out the read duplication with Picard tools (http://broadinstitute.github.io/picard). The open-source DeepVariant (https://github.com/google/&6;deepvariant) (Poplin et al., 2018) tool was used to create individual genome call sets, followed by merging call sets with Bcftools 1.9 (Bcftools by samtools) then analyzing the merged call set. The merged variants were filtered with ‘maf 0.01 min-meanDP 2 minQ 20’. Filtering was done using Vcftools 0.1.16 (Danecek et al., 2011). Retained high-quality sites were used for downstream analysis. Functional annotation of SNPs was done using SnpEff v.4.3 (Cingolani et al., 2012).

### Counting *k-mer*s

**Figure 7:**
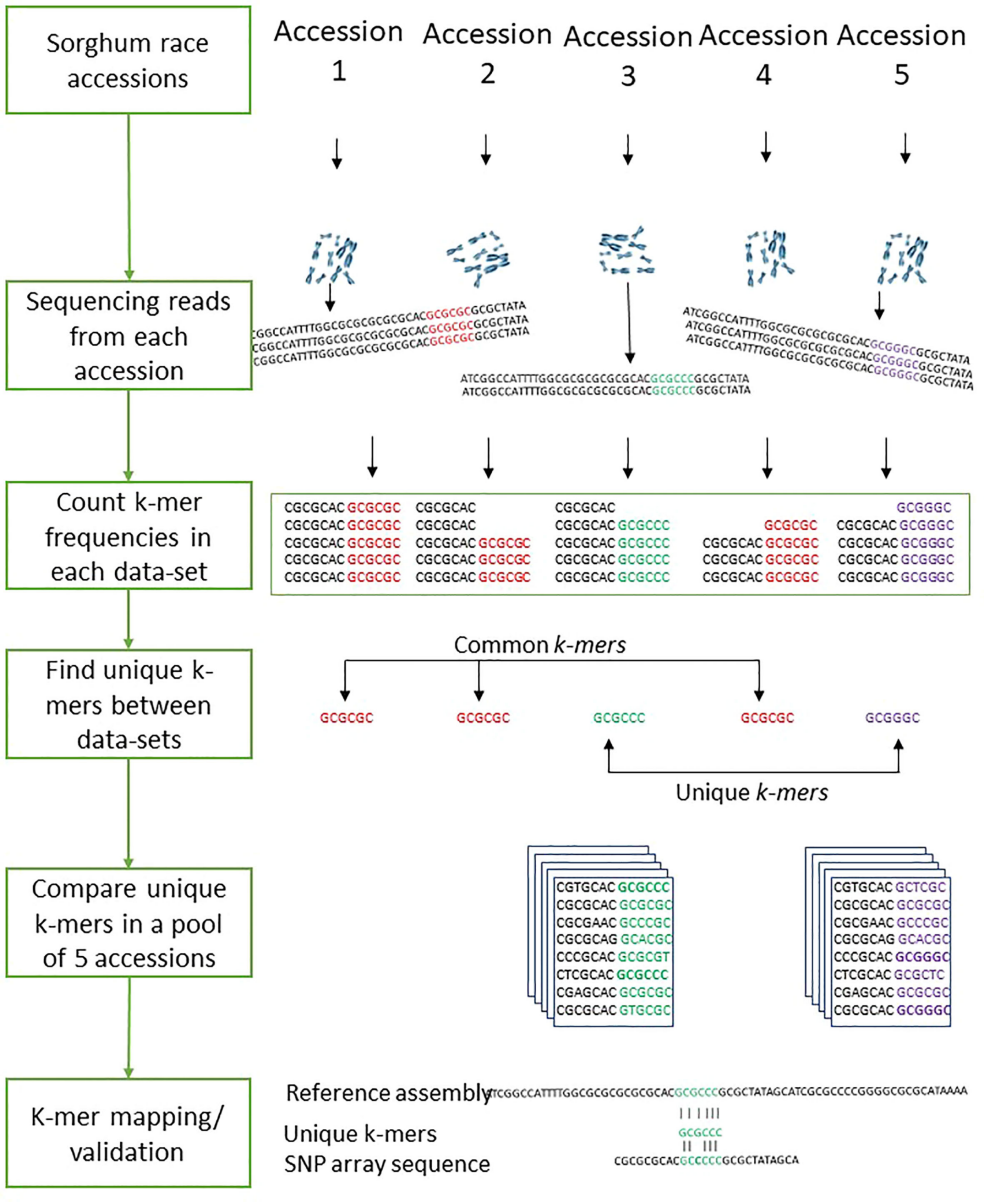
The workflows of the sorghum race accessions comparison with *k-mer* analysis.

The *k-mer-based* genetic distance between 272 sorghum accessions was measured with Mash (Ondov et al., 2016). Out of the 272 accessions used, the mean distance values within each race were used to compare *k-mer*s between the sorghum races. To compare sequences across sorghum races, we determined *k-mer* frequency in sequencing reads from all samples. To identify the common and unique genomic sequences between the sorghum races, we split the sequencing reads into *k* length of the sequence. The optimal *k-mer* size for identifying the distinct *k-mer*s was estimated using KmerGenie (Chikhi and Medvedev, 2014) within the *k* range of 21 to 121. The optimized *k*=47 was used for measuring the *k-mer* frequency as shown in the **Figure 7**. We used the hash-based tool Jellyfish (Marçais and Kingsford, 2011) to count *k-mer*s with the optimized *k-mer* length of 31 (kmer 31, expect number of k-mers 100G, count both strand canonical representation, number of threads 25, number of files open simultaneously 2, output file name) and filtered out *k-mer*s that appeared only once in samples as they were likely from sequencing errors. The *k-mer* hashes were visually inspected through KAT density plots (Mapleson et al., 2017) for all five sorghum race accessions by producing the *k-mer* frequency, GC plots and contamination checks. Unique *k-mer*s mapped to sorghum pangenome were validated with mapped SNParray region from a previous study (Ruperao et al., 2021). Based on the Bowtie2 v2.4.2 (Langmead and Salzberg, 2012) mapped *k-mers*, the gene coverage was assessed with samtools mpileup (Li et al., 2009). The sequence region supporting with minimum of three k-mers was considered as sequence region present in the genome. The gene PAVs were extracted from the sorghum pan-genome genes PAVs catalog (Ruperao et al., 2021) with in-house developed script.

### Nucleotide diversity and relatedness

The filtered SNPs were further subgrouped based on race-specific variant alleles. Nucleotide diversity (*pi*) was calculated using Vcftools 0.1.16 (Danecek et al., 2011). The *pi* (*π*) distributions were compared to assess changes in genetic diversity over time. The *pi* (*π*) density plots were generated with in-house developed scripts.

In addition, a 1,000 bootstrap resampling was used to estimate the genetic relationship among the accessions with R “ape” (Paradis et al., 2004) package to construct a NJ tree and visualized it in iTOL tree viewer (Letunic and Bork, 2019). The Pco analysis was done with R “labdsv” package (https://CRAN.R-project.org/package=labdsv). The admixture v1.3.0 (Alexander et al., 2009) was used to estimate the population structure enabling the cross-validation (CV) with –cv flag. The cross-validation procedure was performed to 10-fold and the lowest CV was considered as optimal K value and the results were visualized with R package (github.com/royfrancis/pophelperShiny) POPHELPER v2.1.1 (Francis, 2017). PopLDdecay (Zhang et al., 2019) was used with MAF 0.01 and MaxDist 2000 to generate the linkage disequilibrium stats and Plot_MultiPop.pl used for plotting the LDdecay.

### Population differentiation and signatures of selection

Tajima’s D (Tajima, 1989) and per-site *Fst* (based on Weir and Cockerham’s *Fst* estimator) (Weir and Cockerham, 1984) were calculated using Vcftools 0.1.16 software (Danecek et al., 2011). Integrated haplotype score (iHS) (Voight et al., 2006) analysis was performed using the “rehh” package (Gautier et al., 2017) in R v 3.6.3, while the extended haplotype-based homozygosity score test) (XP-EHH) (Sabeti et al., 2007) was derived using Beagle (Browning et al., 2021). Significant selective sweeps were detected using the Bonferroni FDR threshold (*P* < 0.05).

Overlap of putative genomic regions under selection with previously known QTLs was detected after downloading the mapped QTL regions from Hostetler et al. (2021) and comparing them with the identified selection regions.

### Access to raw data

We obtained publicly accessible raw Illumina sequence data from three previous studies as shown in **Table 1** and **Supplementary Table 1**. The sorghum accessions having minimum 5x coverage of whole-genome sequence data were used for the analysis, resulting in a total of 272 sorghum accessions.

## Conclusion

This study compared the genomes of the sorghum races with short *k-mer* length sequence to identify the conserved and signature patterns of sorghum race sequences. We implemented a deep learning method to detect the variants and compared structural and functional annotations. On applying the *k-mer*-based genome comparison among the sorghum races, we were able to identify the unique *k-mer* sequences that is specific to the sorghum races and also possibly use as race-specific or accession specific (if *k-mer*s compared between accessions) genetic markers. Our study observed a relatively lower genetic diversity in the caudatum and bicolor races than in kafir, guinea and durra races. Our results revealed several putative footprints of selection that harbor interesting candidate genes associated with agronomically important traits using different statistical approaches. The findings will enhance our understanding of the dynamics of the sorghum race genomes and help to design strategies to breed better genotypes.

## Data availability statement

The datasets presented in this study can be found in online repositories. The names of the repository/repositories and accession number(s) can be found in the article/**Supplementary Material**.

## Author contributions

PR conceived the research. PR, PG, MS, and RD carried out the research. SS managed the computational resource. SD and DO provided inputs on sorghum history and domestication. NT, EH, EM, SM, and BN assessed and provided inputs for manuscript. PR, NT, AR, and DO drafted and edited the manuscript. All authors contributed to the article and approved the submitted version.
