# Supplementary material for: Exploring the sorghum race level diversity utilizing 272 sorghum accessions genomic resources
Source: Front Plant Sci. 2023 Mar 17;14:1143512. doi: 10.3389/fpls.2023.1143512 (PMC10063887; doi:10.3389/fpls.2023.1143512)
Supplement: Supplementary file 1 [file DataSheet_1.pdf]

A

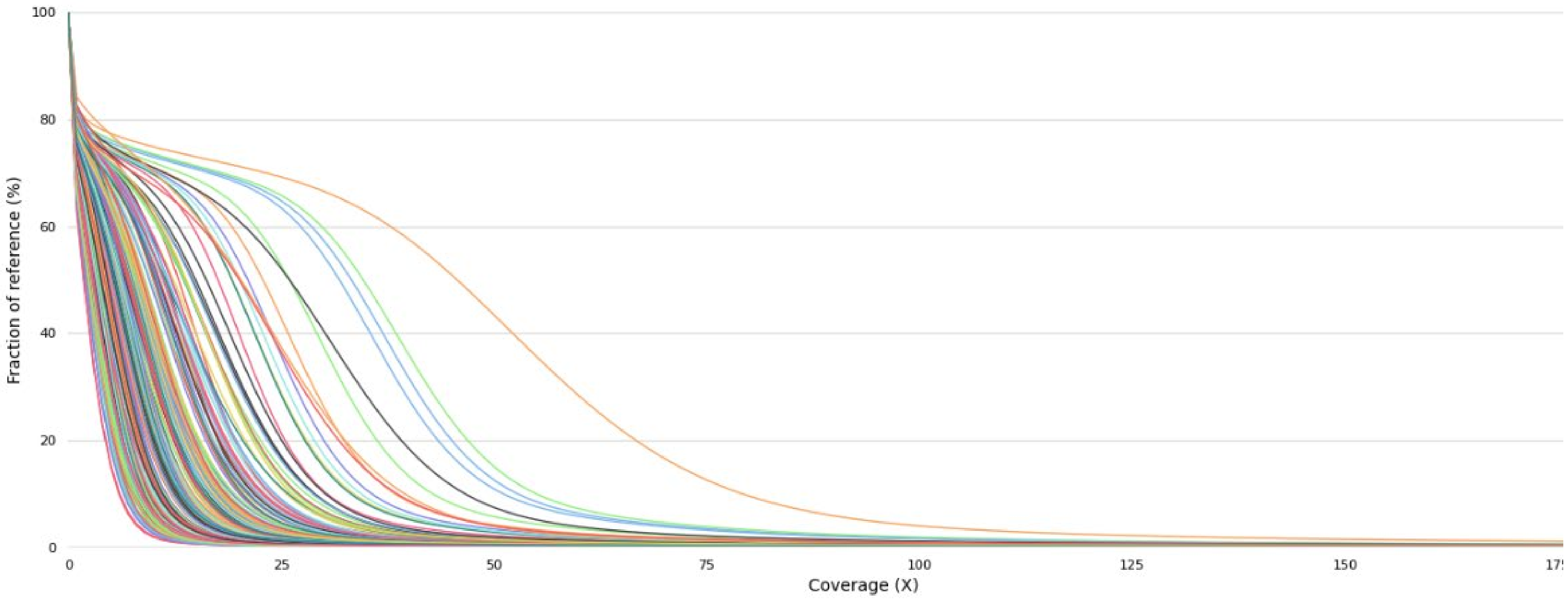
100
80
60
40
20
0
Fraction of reference (%)
0
25
50
75
100
125
150
Coverage (X)

B

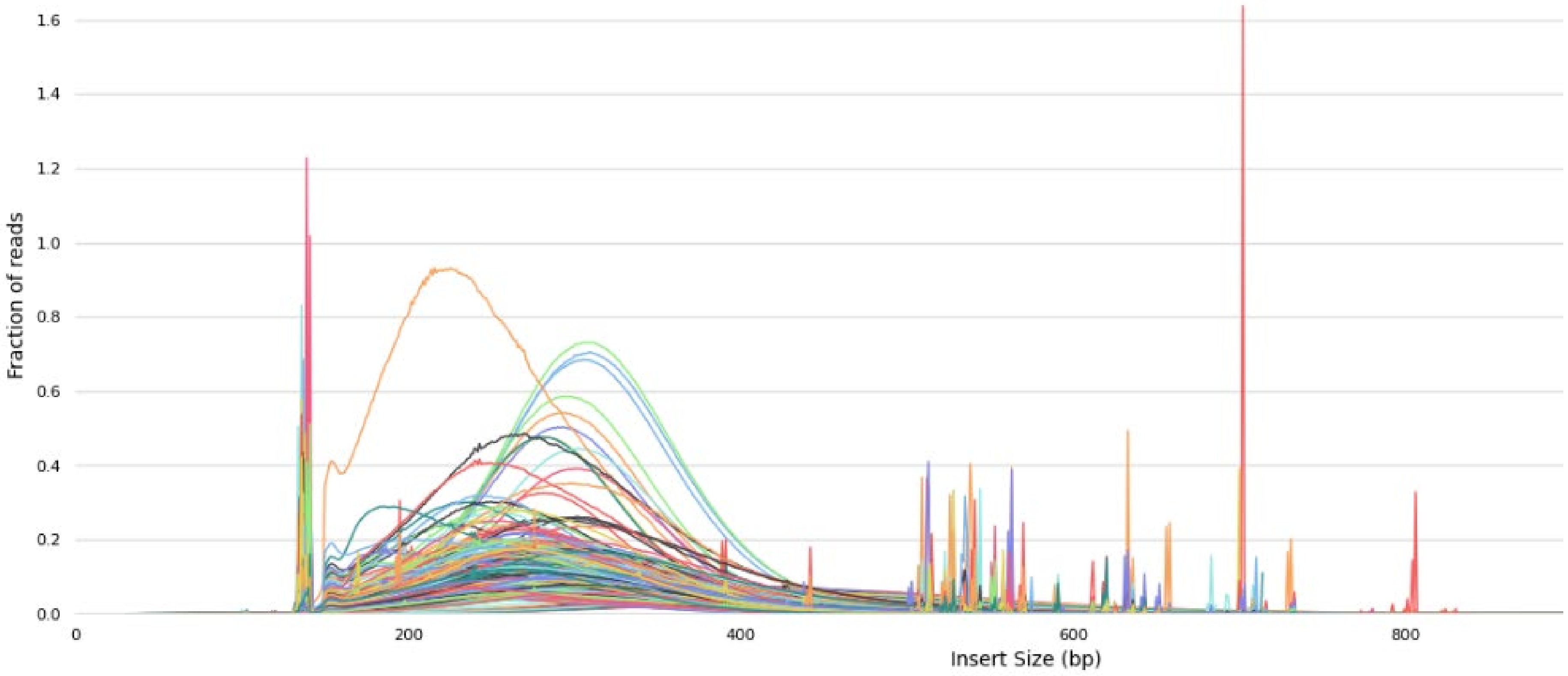
1.6
1.4
1.2
1.0
0.8
0.6
0.4
0.2
0.0
Fraction of reads
0
200
400
600
800
Insert Size (bp)

C

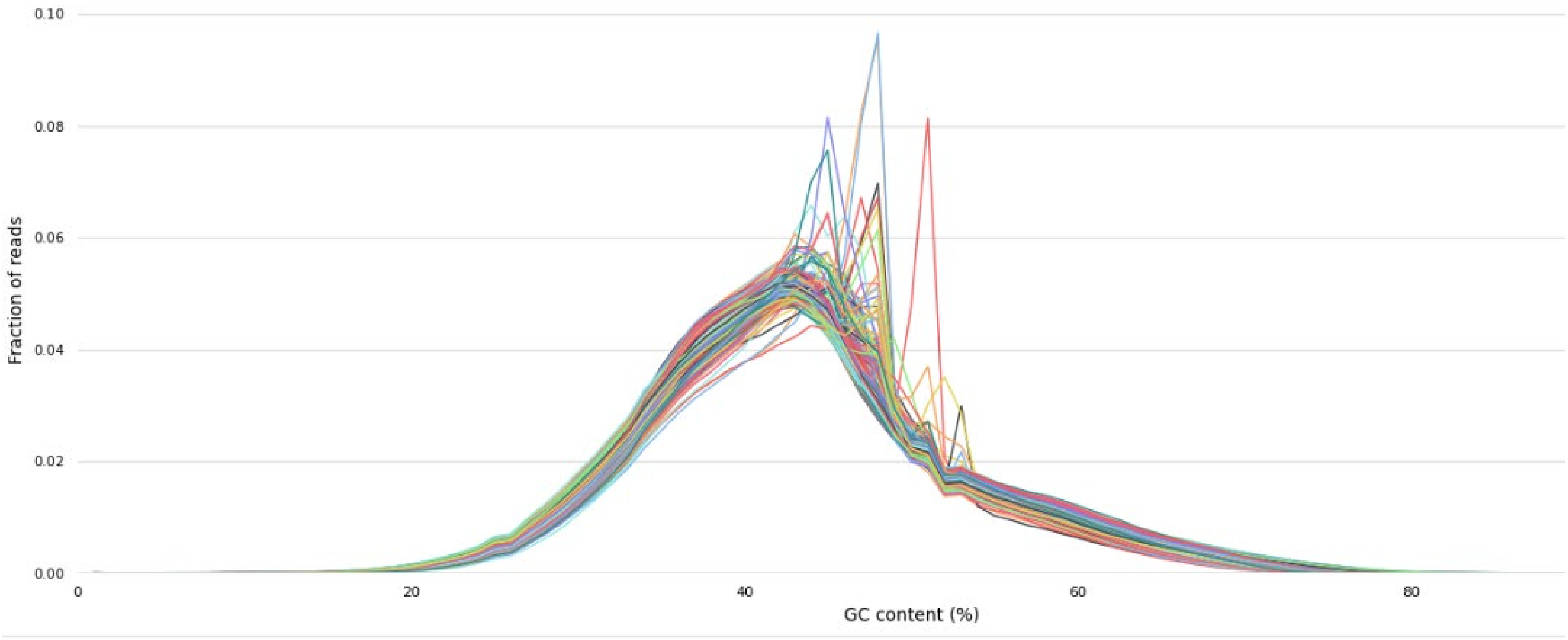

Supplementary Figure 1: Sorghum WGRS mapped stats showing the **A)** reference genome covered in both horizontal and vertical coverage, **B)** inset size of complete datasets **C)** GC content in all sorghum accessions.

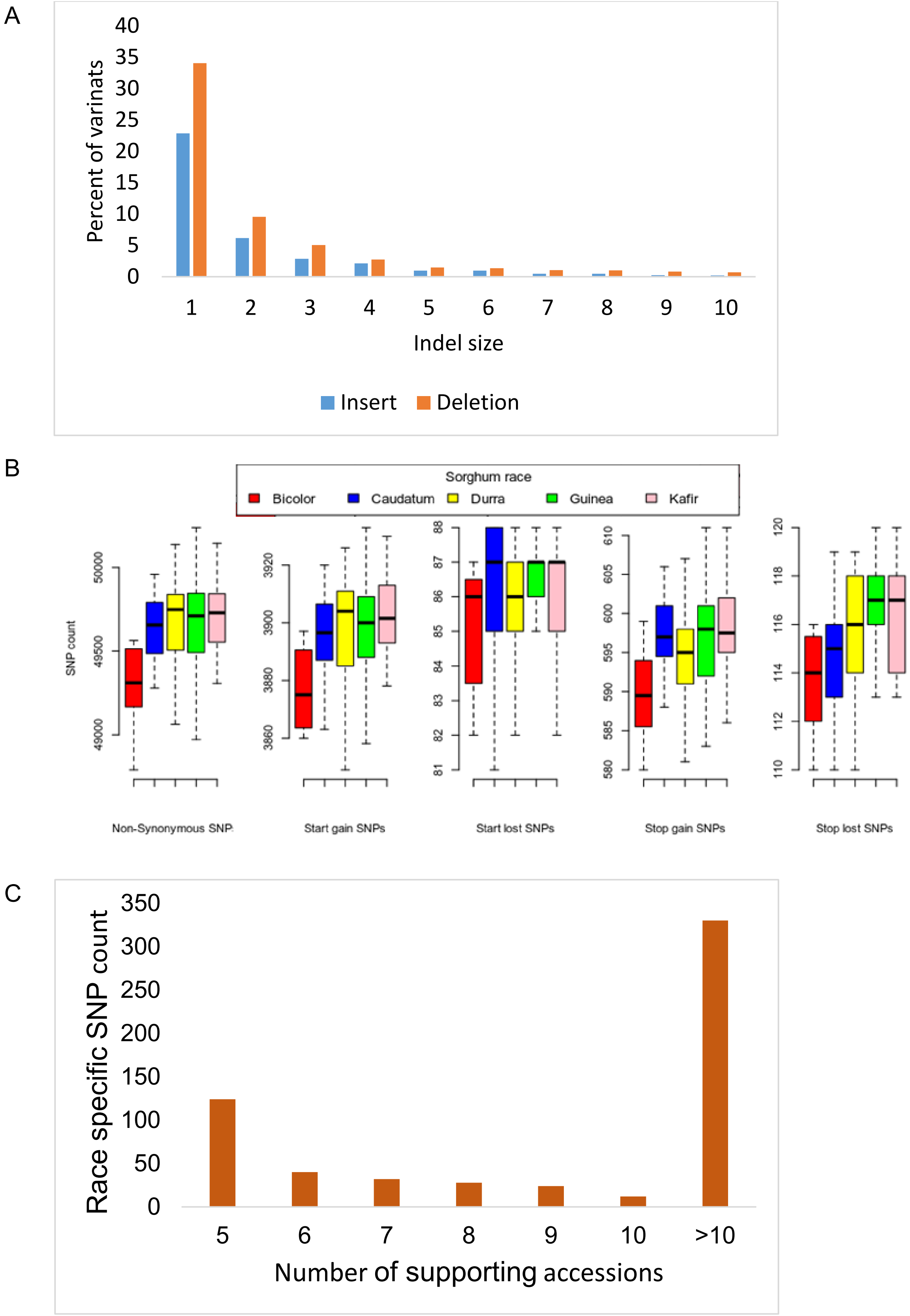

A
40
35
30
25
20
15
10
5
0
Percent of varinats
1
2
3
4
5
6
7
8
9
10
Indel size
Insert
Deletion
B
Sorghum race
Bicolor
Caudatum
Durra
Guinea
Kafir
SNP count
50000
49500
49000
Non-Synonymous SNP
3920
3900
3880
3860
Start gain SNPs
88
87
86
85
84
83
82
81
Start lost SNPs
610
605
600
595
590
585
580
Stop gain SNPs
120
118
116
114
112
110
Stop lost SNPs
C
350
300
250
200
150
100
50
0
Race specific SNP count
5
6
7
8
9
10
>10
Number of supporting accessions

D

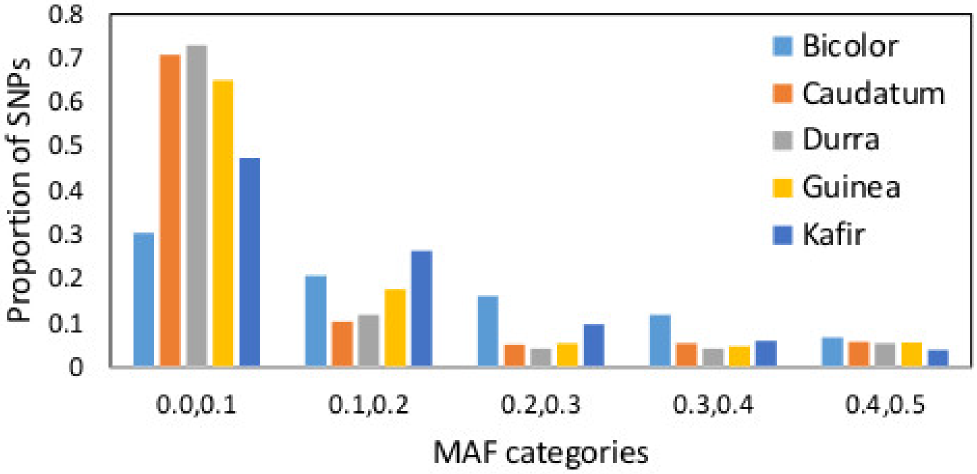

Supplementary Figure 2: Genomic landscape of sorghum. A) Indel length distribution B) SNP annotations C) race-specific SNP calls with a number of supporting sorghum accessions D) The distribution of minor allele frequencies for all sorghum races. SNPs were binned into five categories on the MAF.

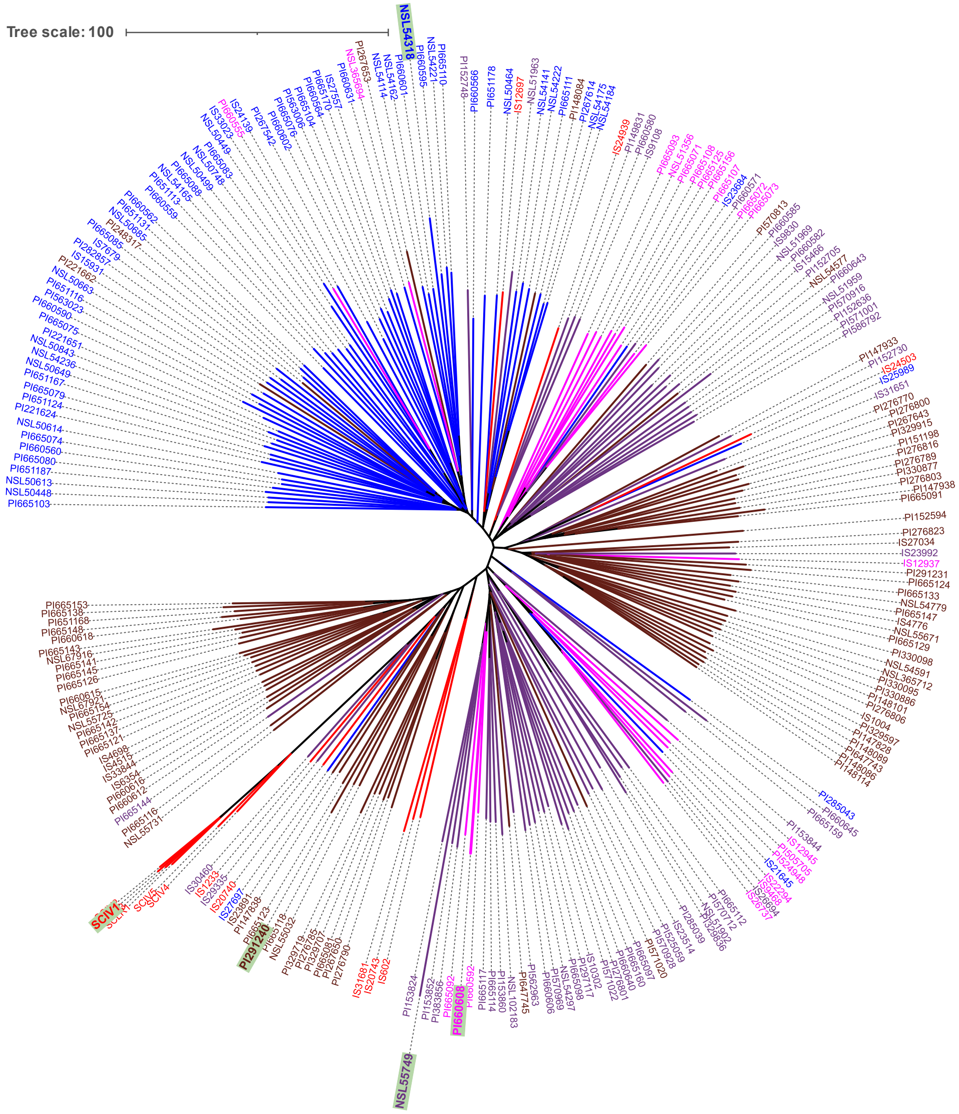

Supplementary Figure 3. Sorghum race accession NJ tree with nodes in blue color are *Guinea*, pink colour accessions are *Kafir*, red color for *bicolor*, brown color in *Durra*, purple color for *Caudatum*. Highlighted node labels are the most distinct accessions in respective sorghum race accessions. The phylogenetic tree contained a distinct cluster of 63 *Guinea* race accessions (nodes in blue colour) mixed with a few other race individuals, like PI221662, PI248317, PI267653 and PI148084 from *Durra* race (nodes in brown colour), PI660555 and NSL365694 from *Kafir* race (nodes in pink colour), IS12697 from *bicolor* race (nodes in red colour). The other sorghum race clusters were split with non-corresponding sorghum race accessions. For example, *Durra* race has 91 accessions split into two clusters with *Caudatum* and *Kafir* race accessions. The sorghum *bicolor* race which was assumed to be more distinct to other race were placed mostly in *Durra* and *Guinea* race cluster. Among the *bicolor* race, the China origin accessions were grouped distinctly in *Durra* cluster compared to other *bicolor* race accessions.

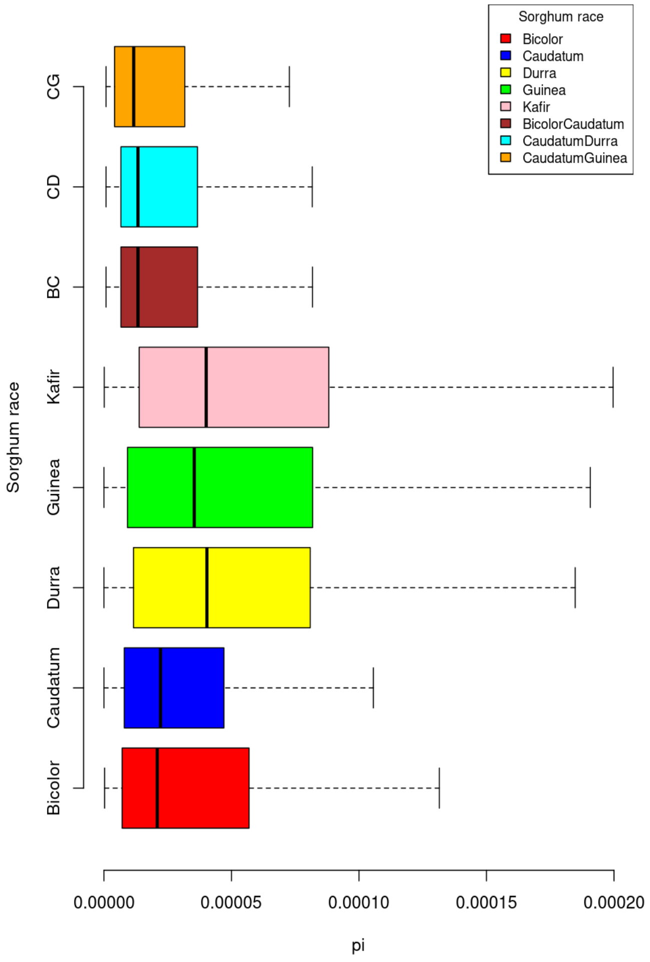

Supplementary Figure 4. The nucleotide diversity comparison between the five sorghum races and three intermediate races.

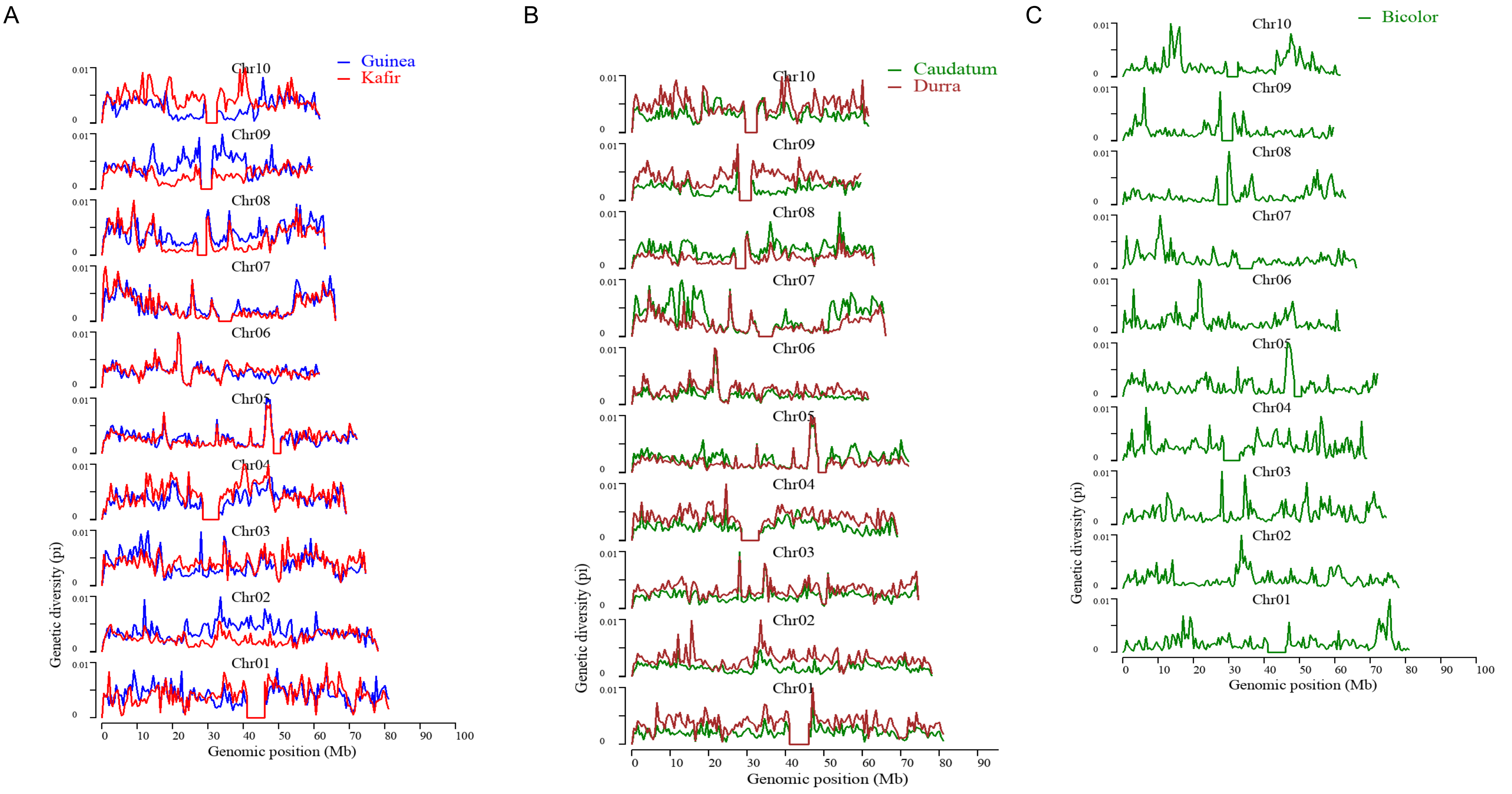

Supplementary Figure 5. Nucleotide diversity (π) in sorghum race populations **A)** Guinea and kafir **B)** Caudatum and Durra **C)** Bicolor

A

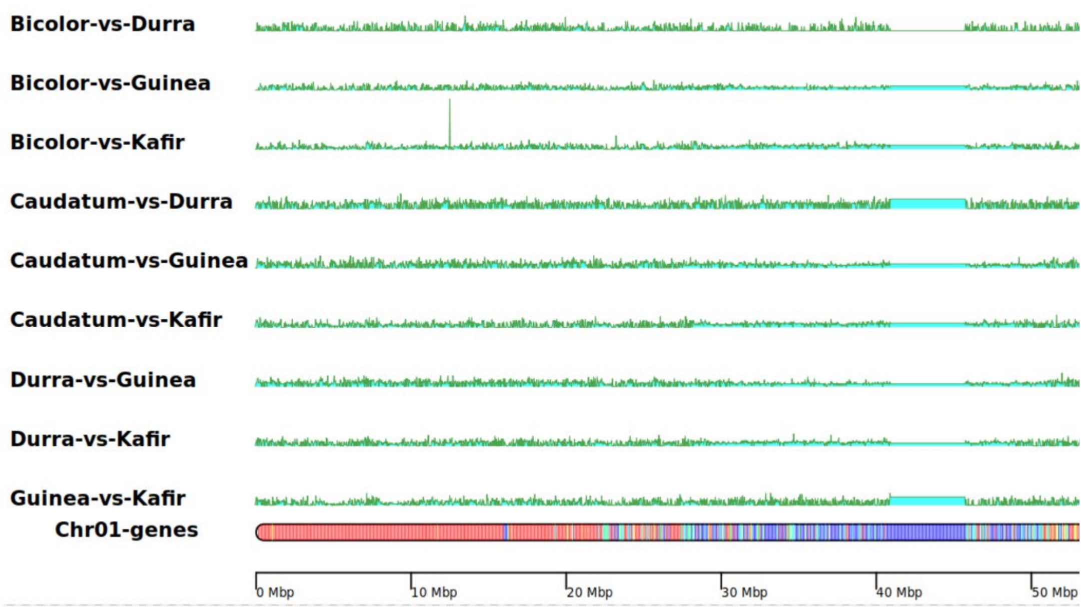
Bicolor-vs-Durra
Bicolor-vs-Guinea
Bicolor-vs-Kafir
Caudatum-vs-Durra
Caudatum-vs-Guinea
Caudatum-vs-Kafir
Durra-vs-Guinea
Durra-vs-Kafir
Guinea-vs-Kafir
Chr01-genes
0 Mbp
10 Mbp
20 Mbp
30 Mbp
40 Mbp
50 Mbp

B

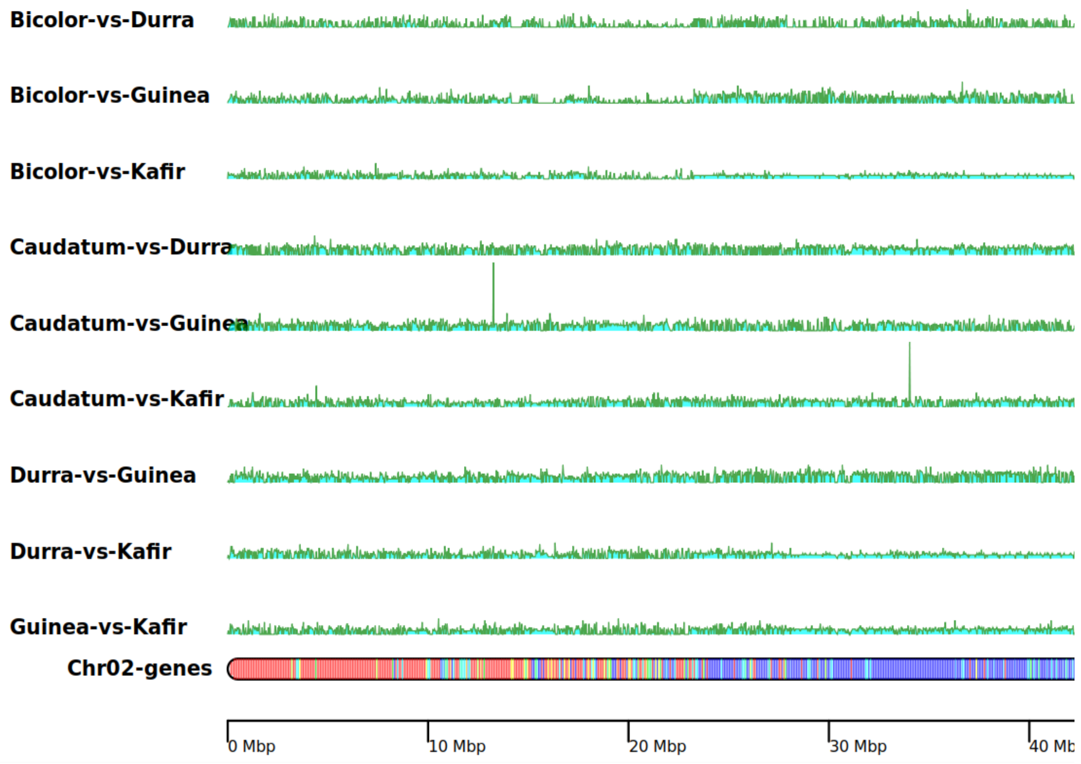
Bicolor-vs-Durra
Bicolor-vs-Guinea
Bicolor-vs-Kafir
Caudatum-vs-Durra
Caudatum-vs-Guinea
Caudatum-vs-Kafir
Durra-vs-Guinea
Durra-vs-Kafir
Guinea-vs-Kafir
Chr02-genes
0 Mbp
10 Mbp
20 Mbp
30 Mbp

C

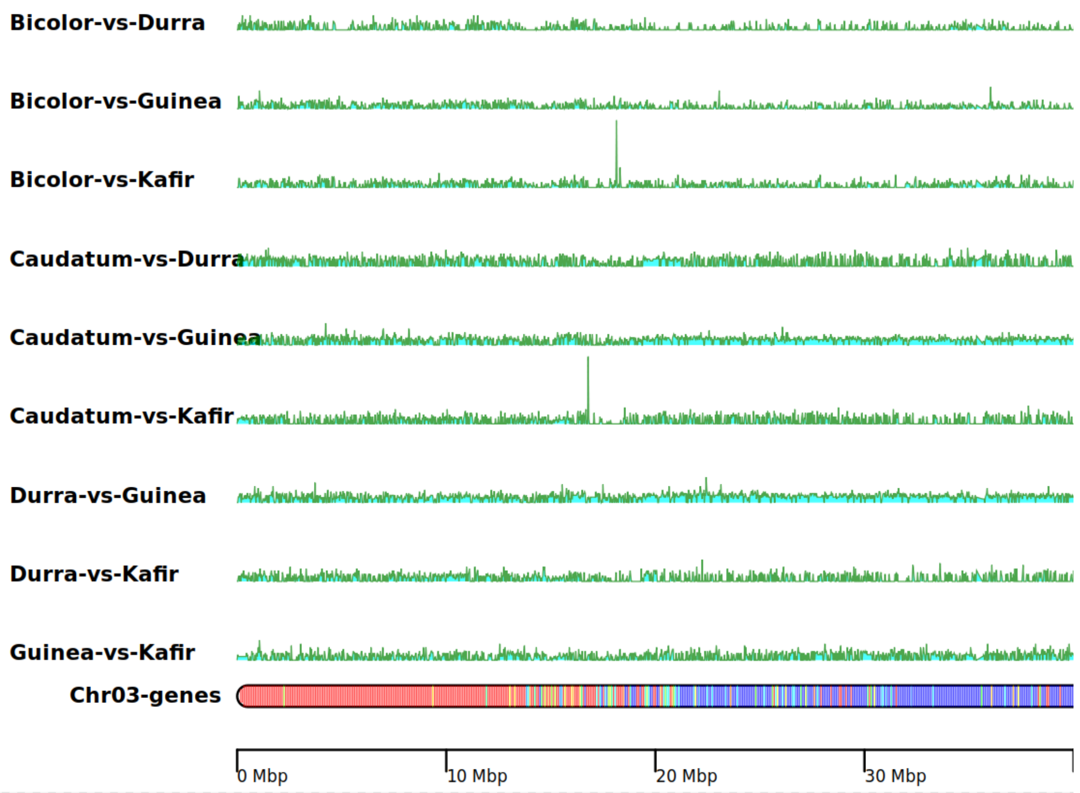
Bicolor-vs-Durra
Bicolor-vs-Guinea
Bicolor-vs-Kafir
Caudatum-vs-Durra
Caudatum-vs-Guinea
Caudatum-vs-Kafir
Durra-vs-Guinea
Durra-vs-Kafir
Guinea-vs-Kafir
Chr03-genes
0 Mbp
10 Mbp
20 Mbp
30 Mbp

D

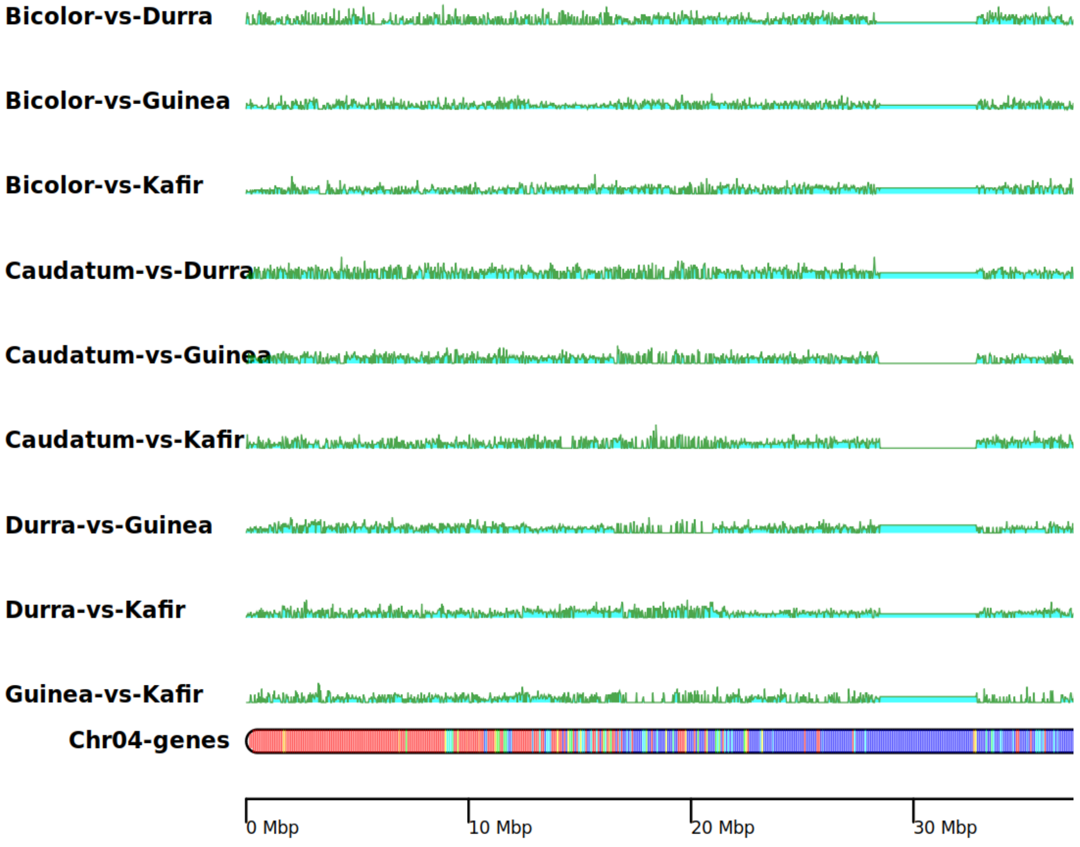
Bicolor-vs-Durra
Bicolor-vs-Guinea
Bicolor-vs-Kafir
Caudatum-vs-Durra
Caudatum-vs-Guinea
Caudatum-vs-Kafir
Durra-vs-Guinea
Durra-vs-Kafir
Guinea-vs-Kafir
Chr04-genes
0 Mbp
10 Mbp
20 Mbp
30 Mbp

E

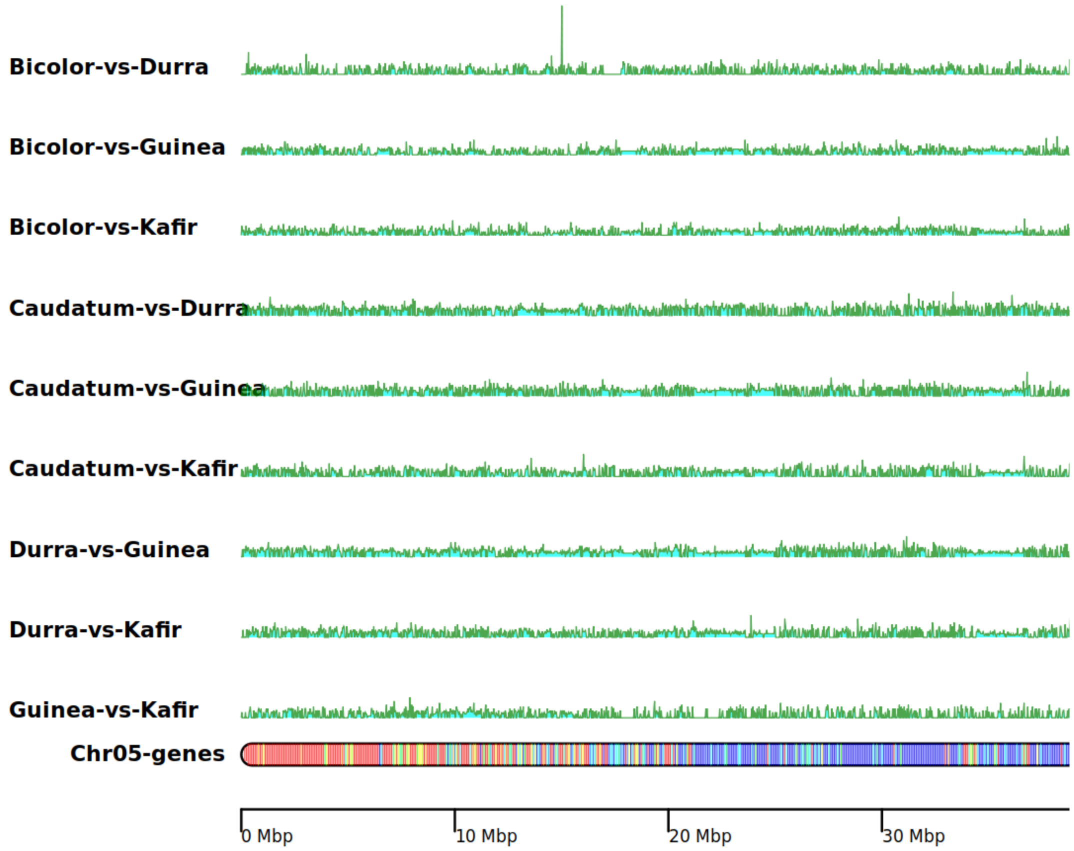
Bicolor-vs-Durra
Bicolor-vs-Guinea
Bicolor-vs-Kafir
Caudatum-vs-Durra
Caudatum-vs-Guinea
Caudatum-vs-Kafir
Durra-vs-Guinea
Durra-vs-Kafir
Guinea-vs-Kafir
Chr05-genes
0 Mbp
10 Mbp
20 Mbp
30 Mbp

F

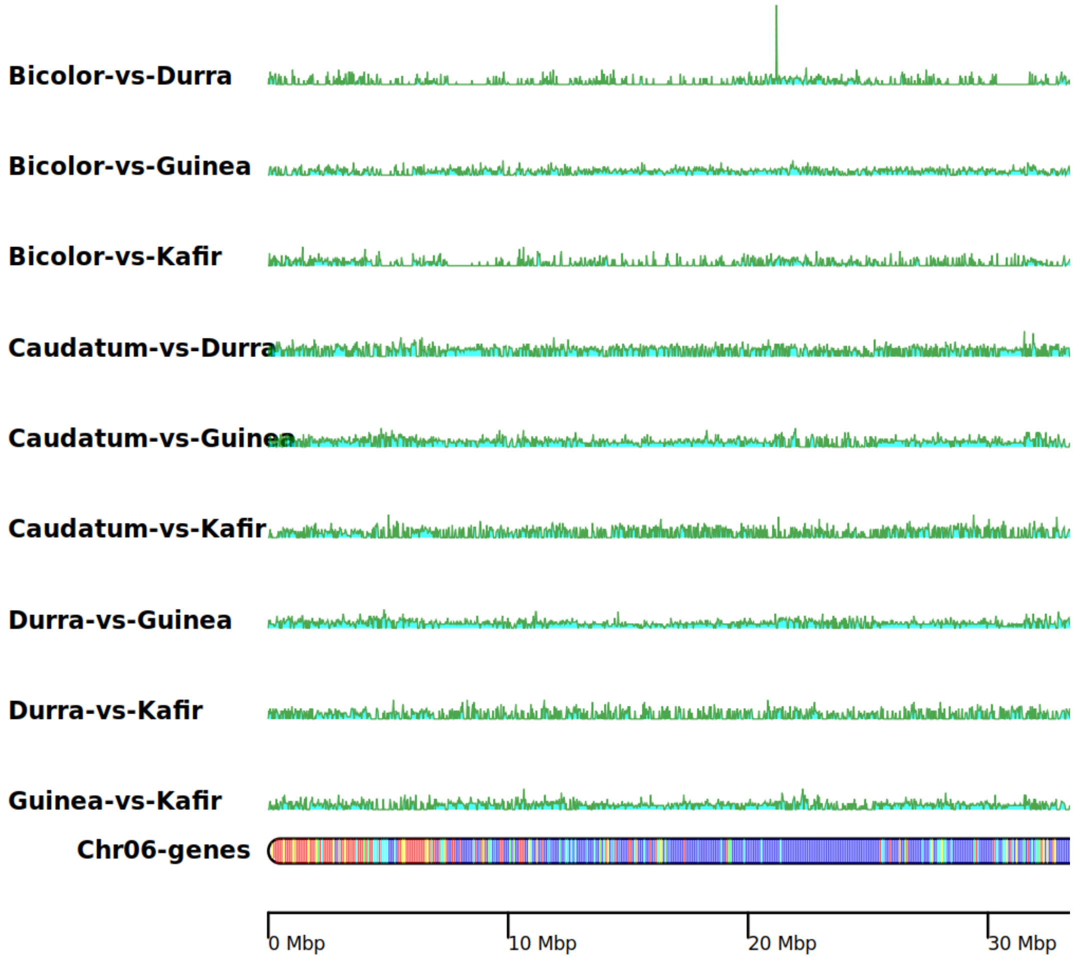

Bicolor-vs-Durra
Bicolor-vs-Guinea
Bicolor-vs-Kafir
Caudatum-vs-Durra
Caudatum-vs-Guinea
Caudatum-vs-Kafir
Durra-vs-Guinea
Durra-vs-Kafir
Guinea-vs-Kafir
Chr06-genes
0 Mbp
10 Mbp
20 Mbp
30 Mbp

G

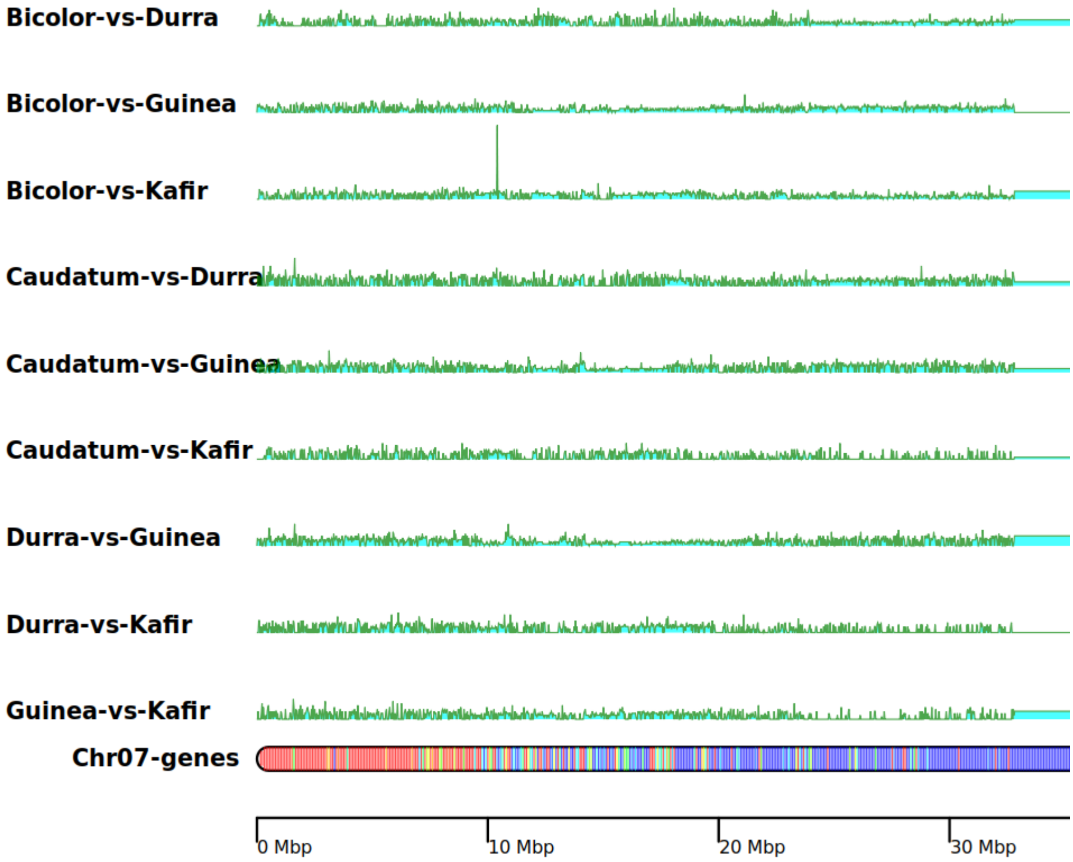
Bicolor-vs-Durra
Bicolor-vs-Guinea
Bicolor-vs-Kafir
Caudatum-vs-Durra
Caudatum-vs-Guinea
Caudatum-vs-Kafir
Durra-vs-Guinea
Durra-vs-Kafir
Guinea-vs-Kafir
Chr07-genes
0 Mbp
10 Mbp
20 Mbp
30 Mbp

H

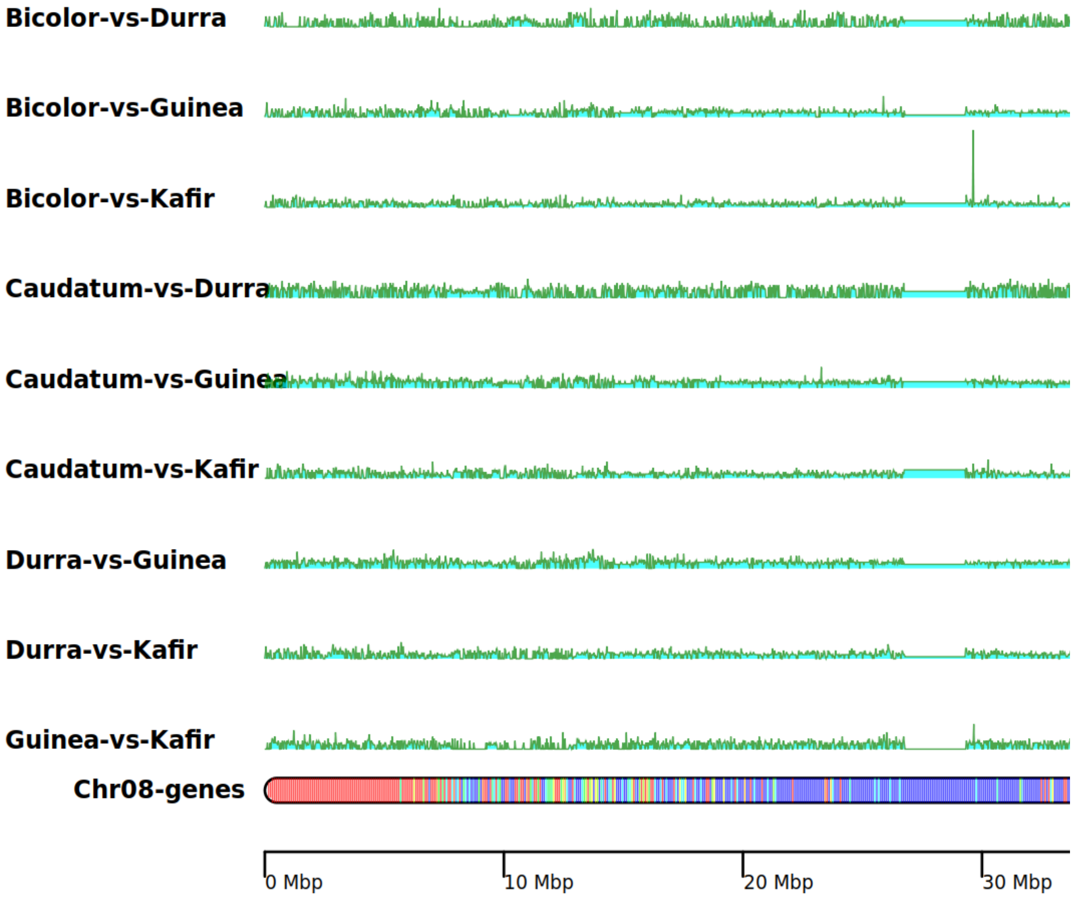
Bicolor-vs-Durra
Bicolor-vs-Guinea
Bicolor-vs-Kafir
Caudatum-vs-Durra
Caudatum-vs-Guinea
Caudatum-vs-Kafir
Durra-vs-Guinea
Durra-vs-Kafir
Guinea-vs-Kafir
Chr08-genes
0 Mbp
10 Mbp
20 Mbp
30 Mbp

I

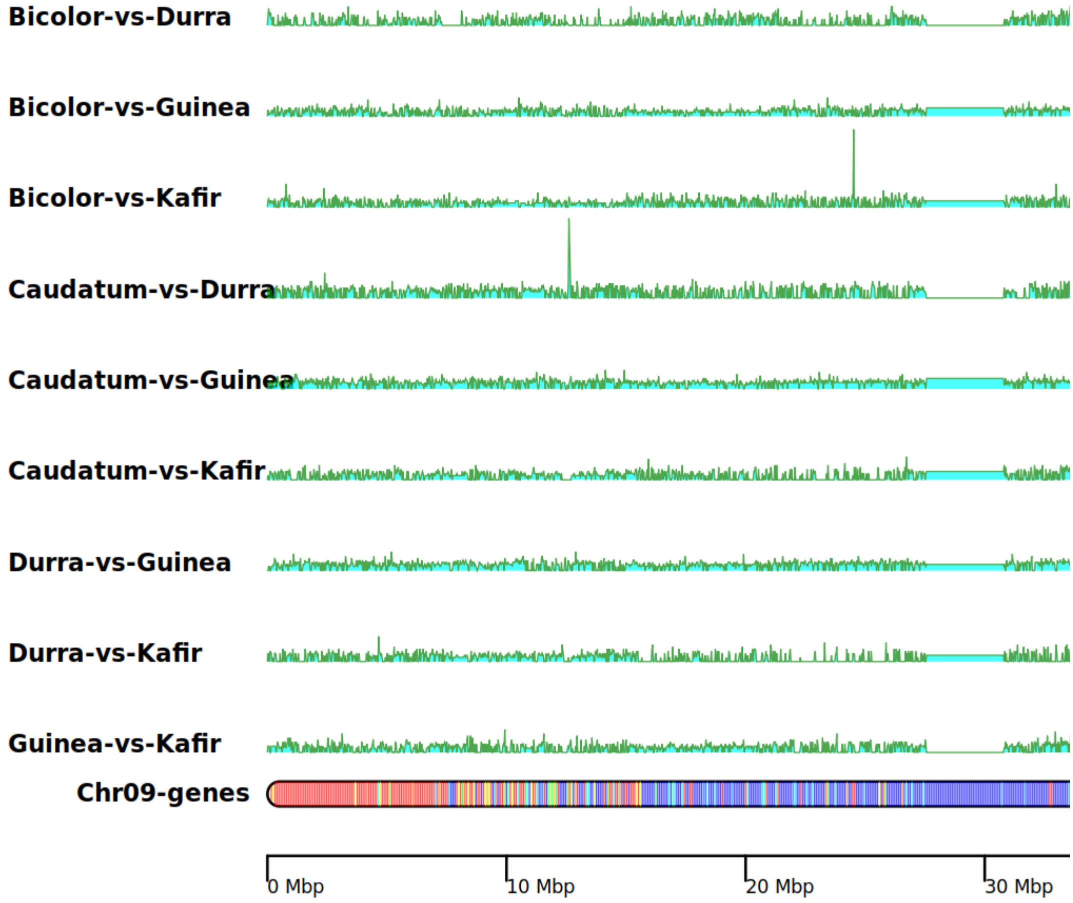
Bicolor-vs-Durra
Bicolor-vs-Guinea
Bicolor-vs-Kafir
Caudatum-vs-Durra
Caudatum-vs-Guinea
Caudatum-vs-Kafir
Durra-vs-Guinea
Durra-vs-Kafir
Guinea-vs-Kafir
Chr09-genes
0 Mbp
10 Mbp
20 Mbp
30 Mbp

J

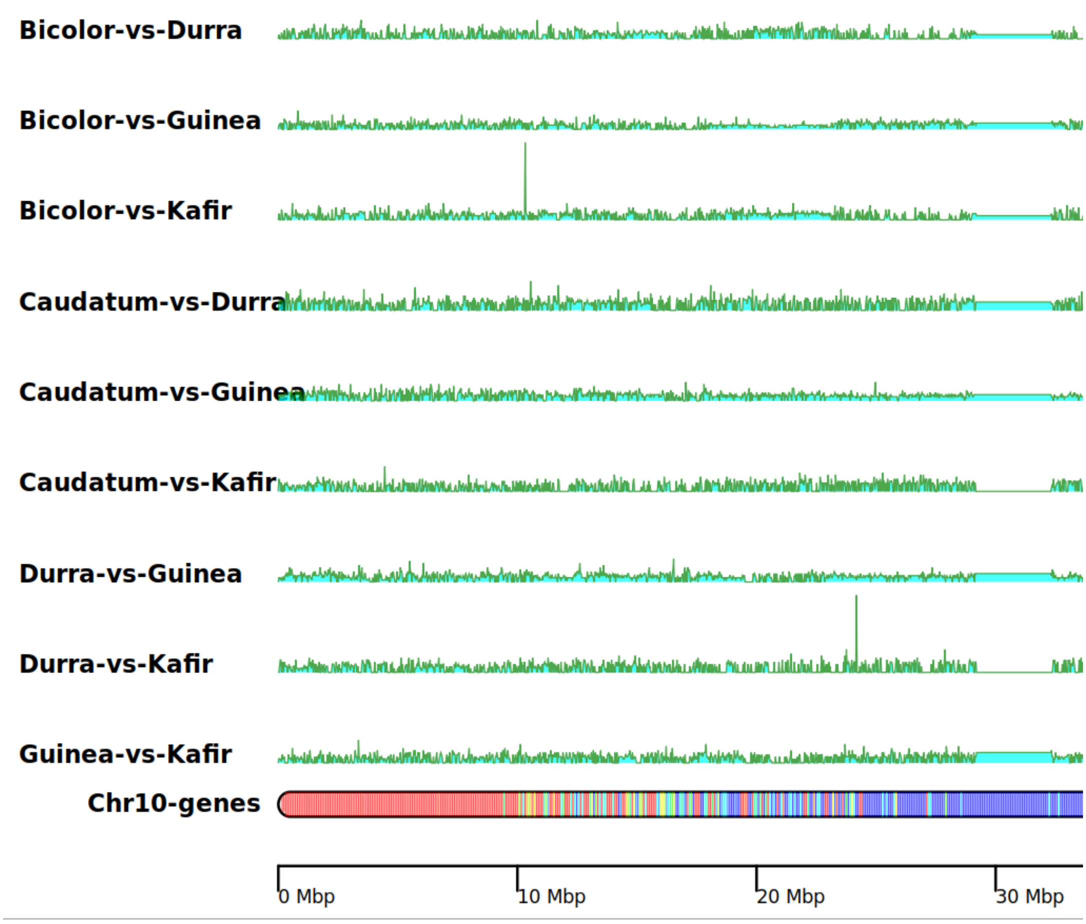

Supplementary Figure 6. Pairwise F_{ST} across the chromosomes of sorghum race populations and gene density heatmap on respective chromosomes.

A

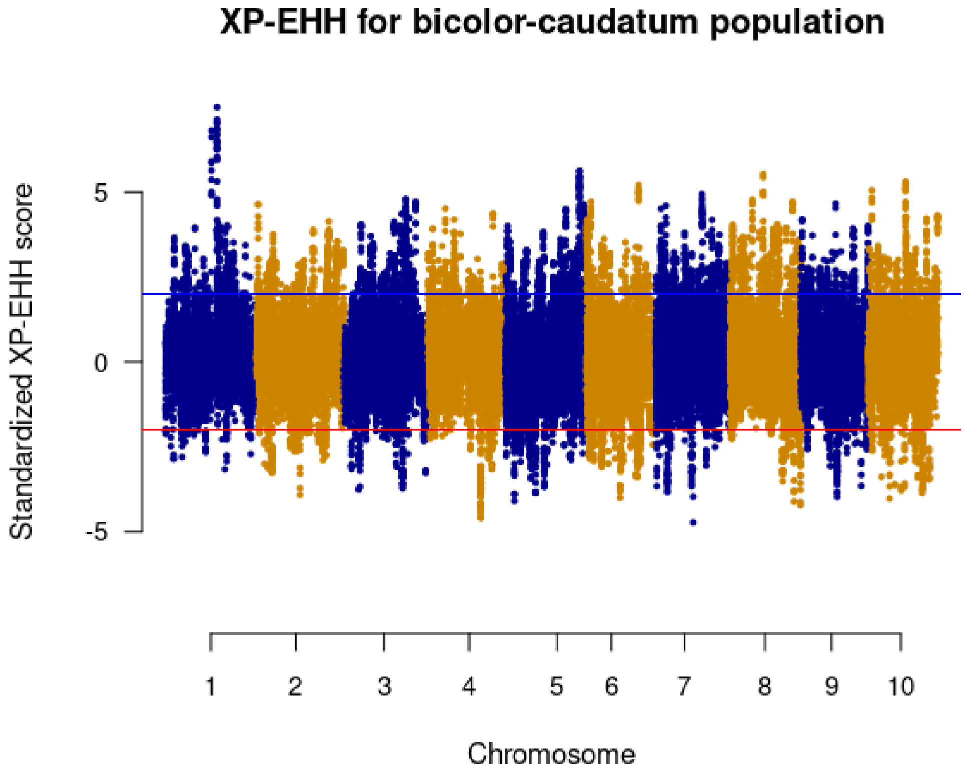
XP-EHH for bicolor-caudatum population
Standardized XP-EHH score
5
0
-5
1
2
3
4
5
6
7
8
9
10
Chromosome

B

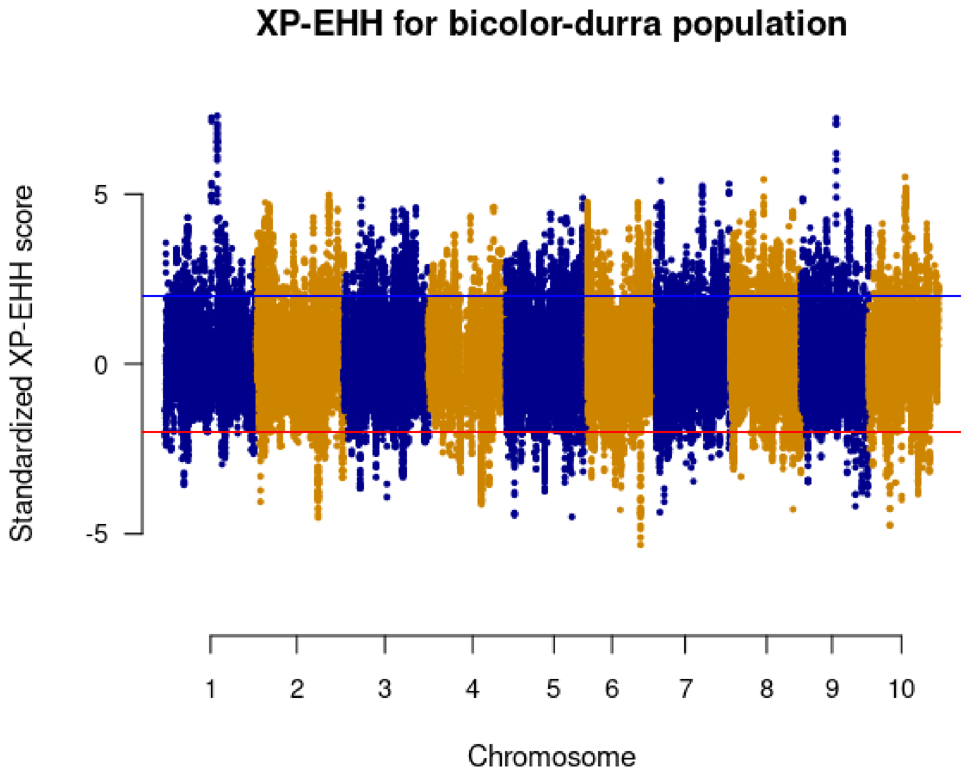
XP-EHH for bicolor-durra population
Standardized XP-EHH score
5
0
-5
1
2
3
4
5
6
7
8
9
10
Chromosome

C

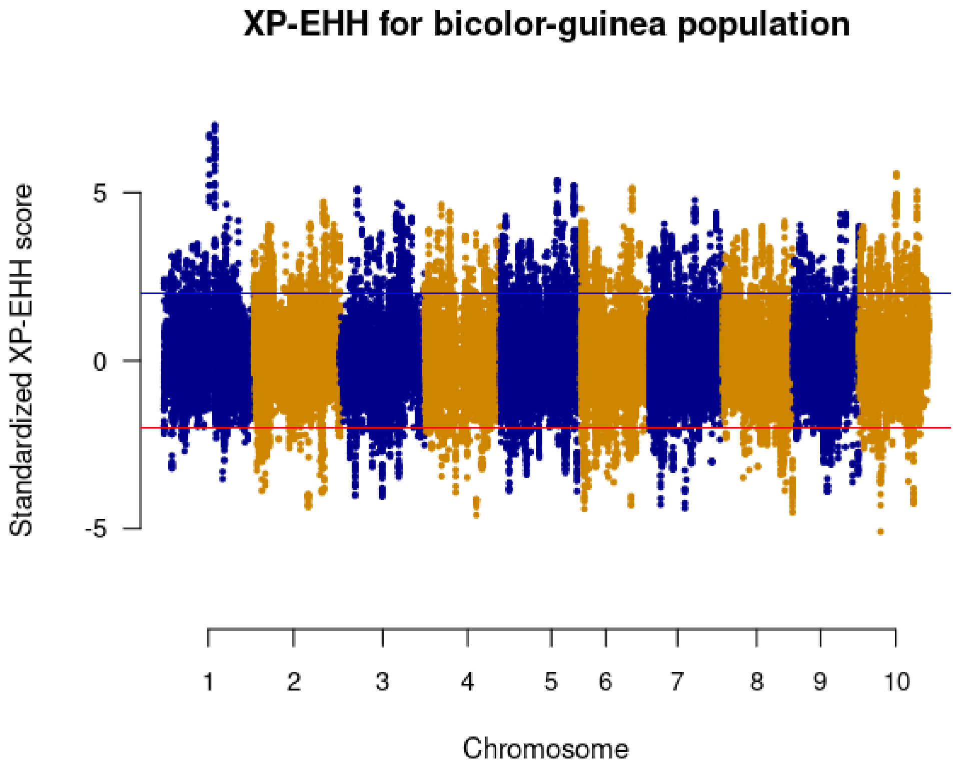
XP-EHH for bicolor-guinea population
Standardized XP-EHH score
5
0
-5
1
2
3
4
5
6
7
8
9
10
Chromosome

D

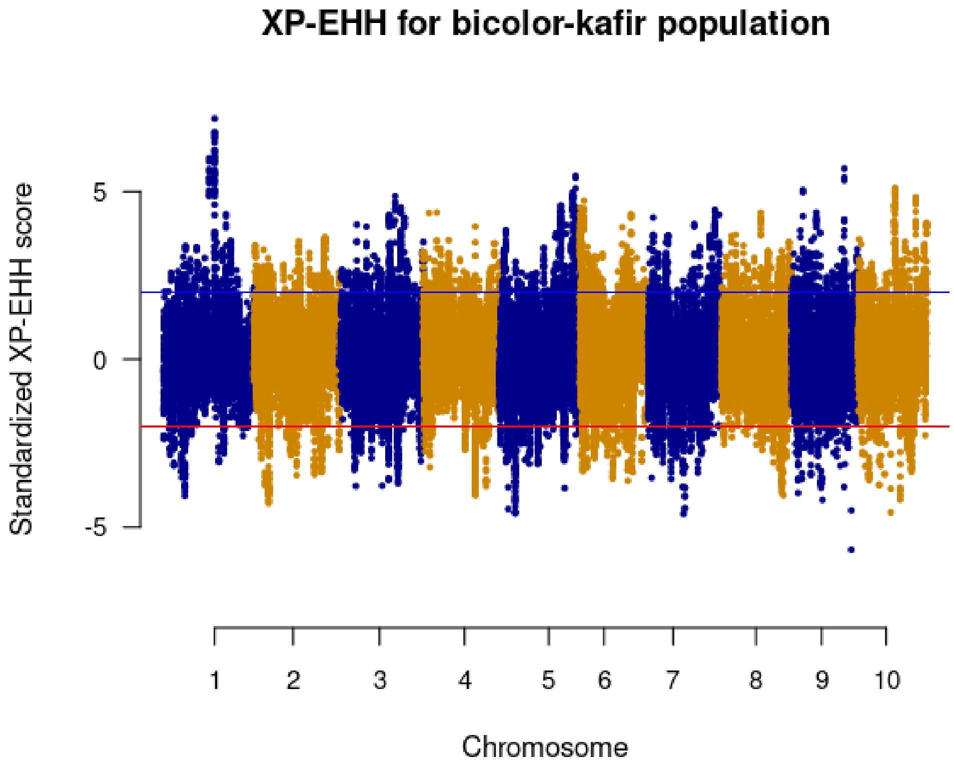
XP-EHH for bicolor-kafir population
Standardized XP-EHH score
5
0
-5
1
2
3
4
5
6
7
8
9
10
Chromosome

E

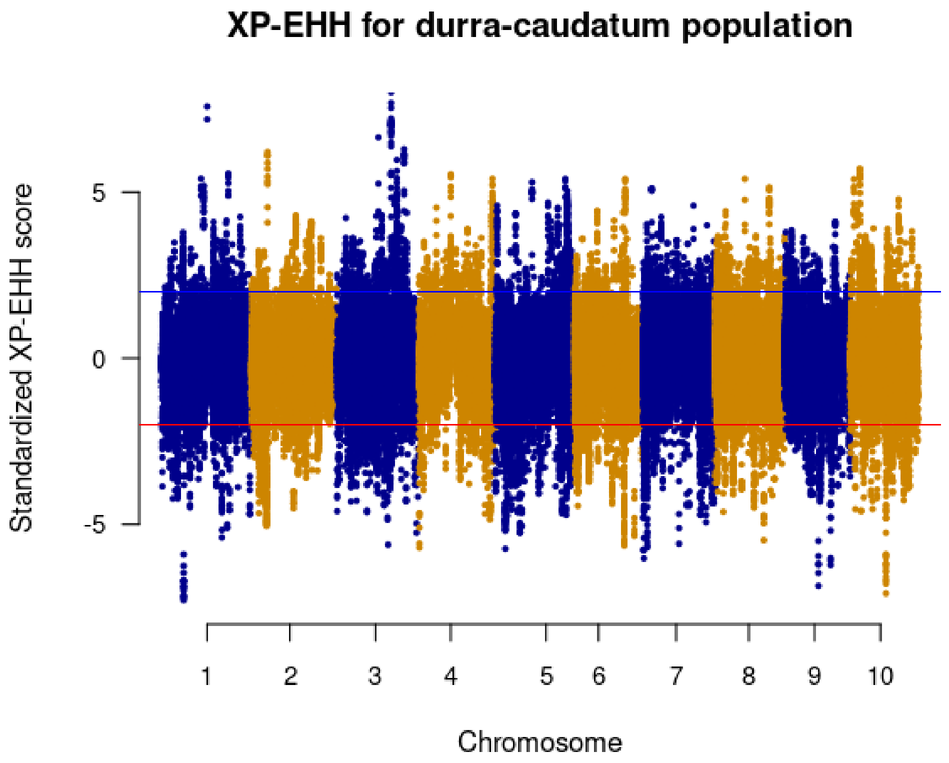
XP-EHH for durra-caudatum population
Standardized XP-EHH score
5
0
-5
1
2
3
4
5
6
7
8
9
10
Chromosome

F

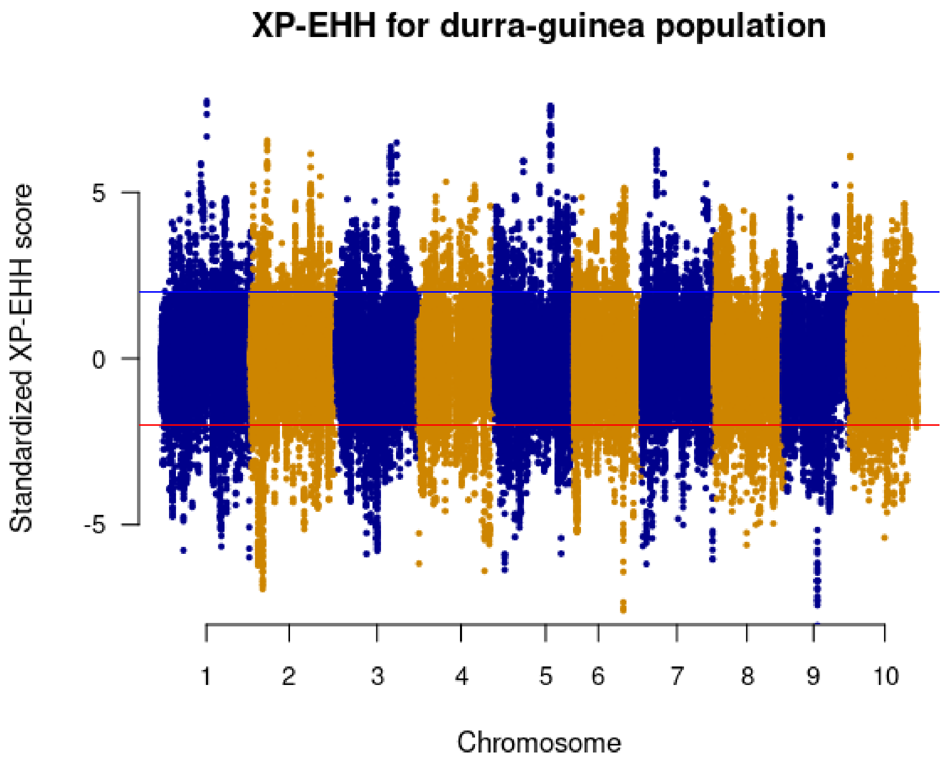
XP-EHH for durra-guinea population
Standardized XP-EHH score
5
0
-5
1
2
3
4
5
6
7
8
9
10
Chromosome

G

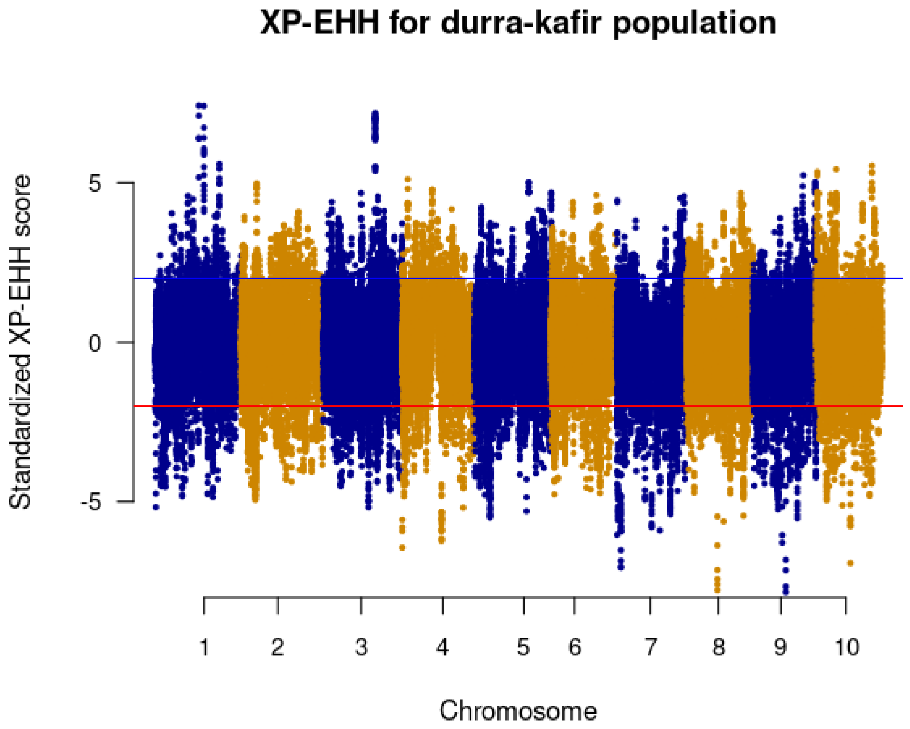
XP-EHH for durra-kafir population
Standardized XP-EHH score
5
0
-5
1
2
3
4
5
6
7
8
9
10
Chromosome

H

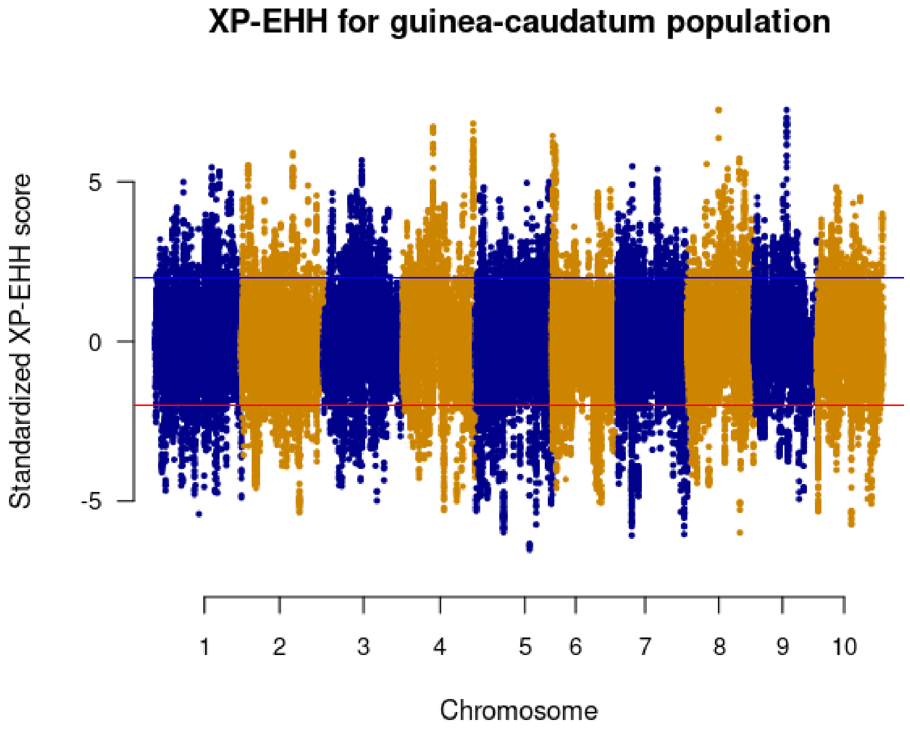
XP-EHH for guinea-caudatum population
Standardized XP-EHH score
5
0
-5
1
2
3
4
5
6
7
8
9
10
Chromosome

I

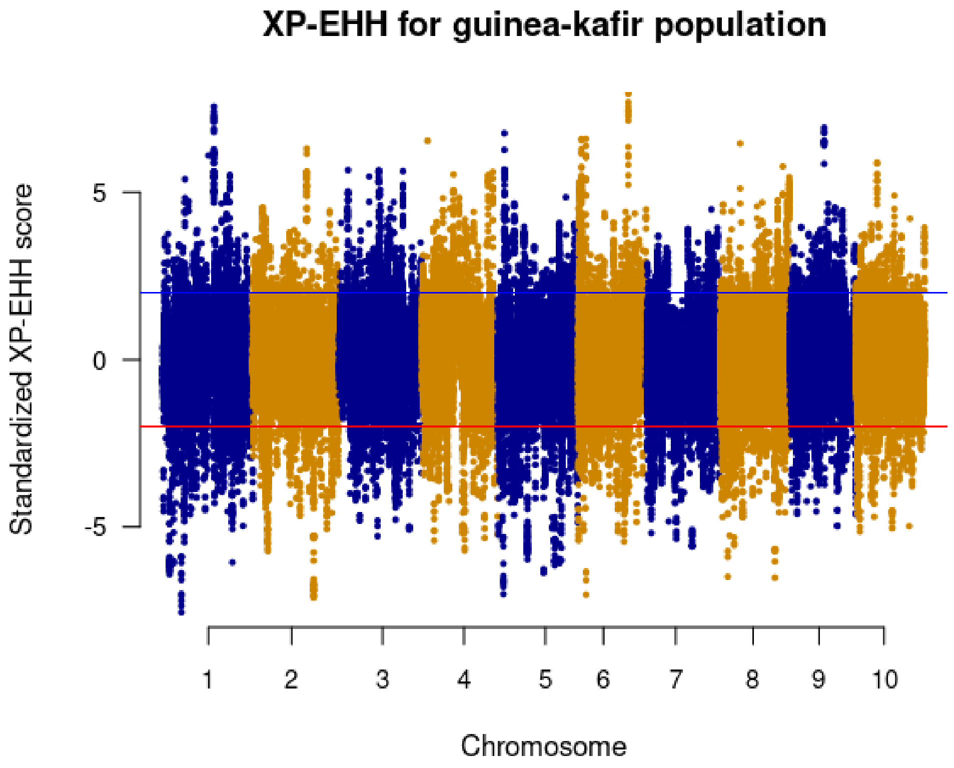

J

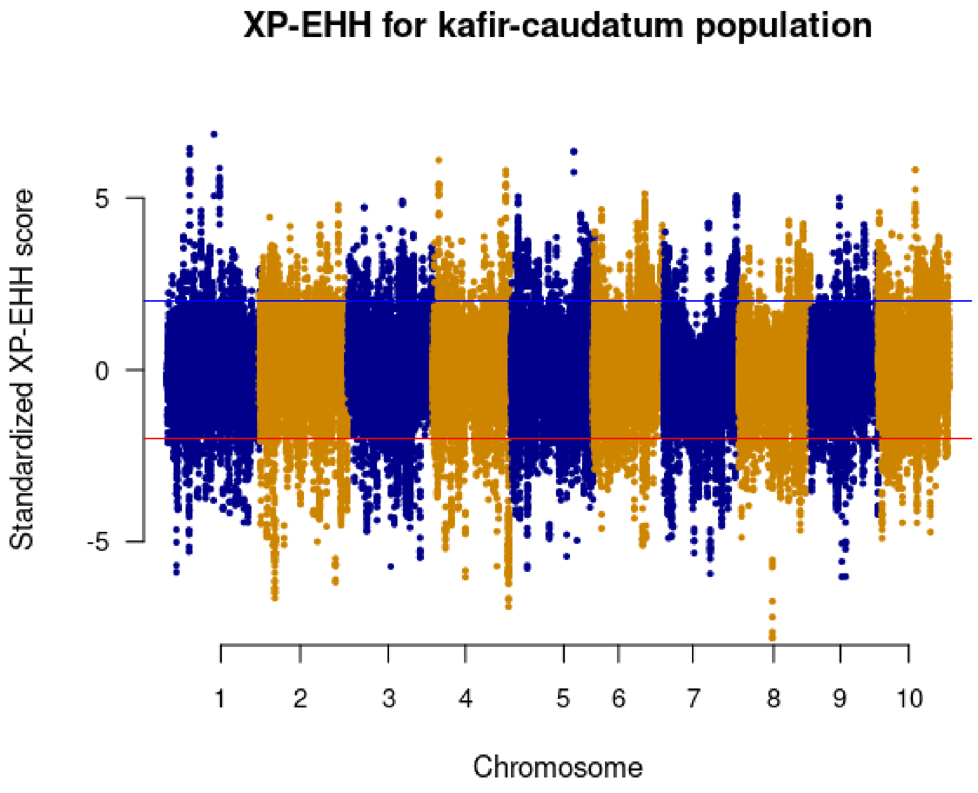

Supplementary Figure 7. Distribution of standardized XP-EHH scores in the sorghum race population comparison

A

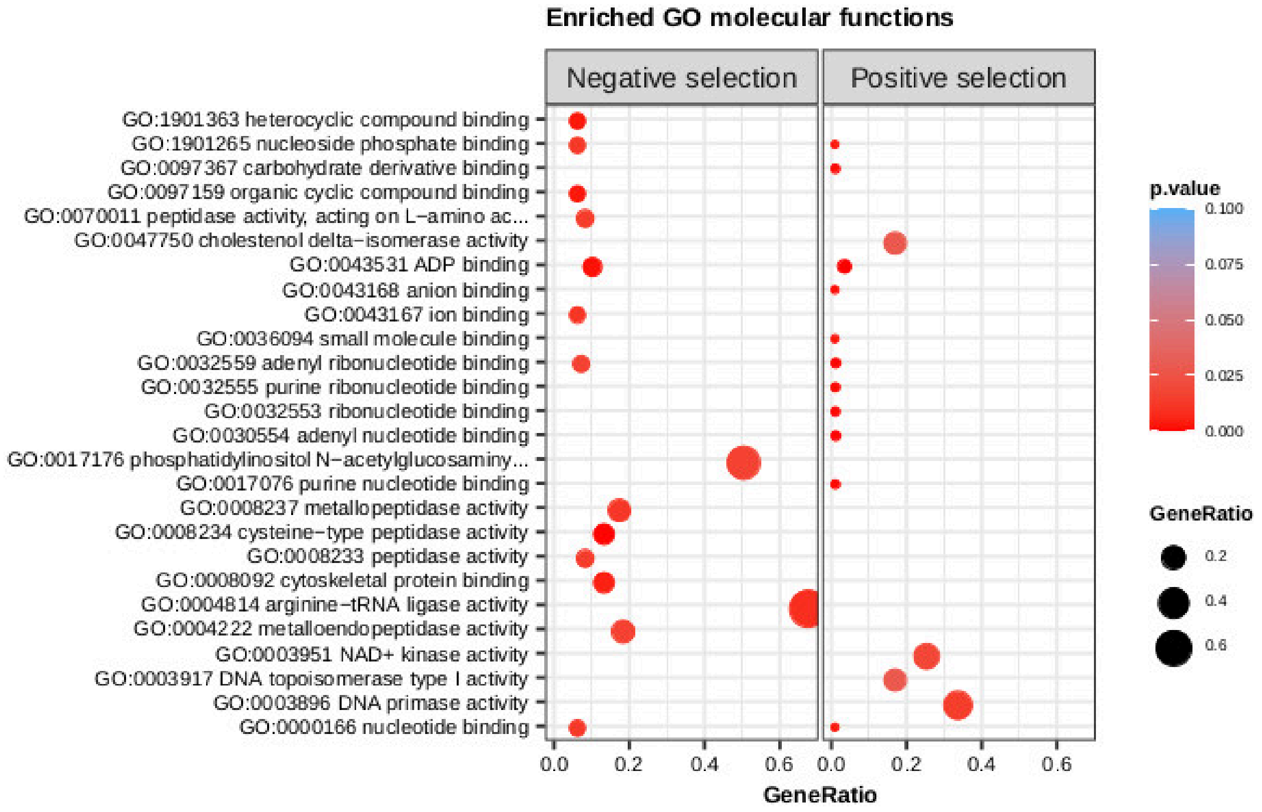
Enriched GO molecular functions
Negative selection
Positive selection
GO:1901363 heterocyclic compound binding
GO:1901265 nucleoside phosphate binding
GO:0097367 carbohydrate derivative binding
GO:0097159 organic cyclic compound binding
GO:0070011 peptidase activity, acting on L−amino ac...
GO:0047750 cholestenol delta−isomerase activity
GO:0043531 ADP binding
GO:0043168 anion binding
GO:0043167 ion binding
GO:0036094 small molecule binding
GO:0032559 adenyl ribonucleotide binding
GO:0032555 purine ribonucleotide binding
GO:0032553 ribonucleotide binding
GO:0030554 adenyl nucleotide binding
GO:0017176 phosphatidylinositol N−acetylglucosaminy...
GO:0017076 purine nucleotide binding
GO:0008237 metallopeptidase activity
GO:0008234 cysteine−type peptidase activity
GO:0008233 peptidase activity
GO:0008092 cytoskeletal protein binding
GO:0004814 arginine−tRNA ligase activity
GO:0004222 metalloendopeptidase activity
GO:0003951 NAD+ kinase activity
GO:0003917 DNA topoisomerase type I activity
GO:0003896 DNA primase activity
GO:0000166 nucleotide binding
0.0 0.2 0.4 0.6 0.0 0.2 0.4 0.6
GeneRatio
p.value
0.100
0.075
0.050
0.025
0.000
GeneRatio
0.2
0.4
0.6

B

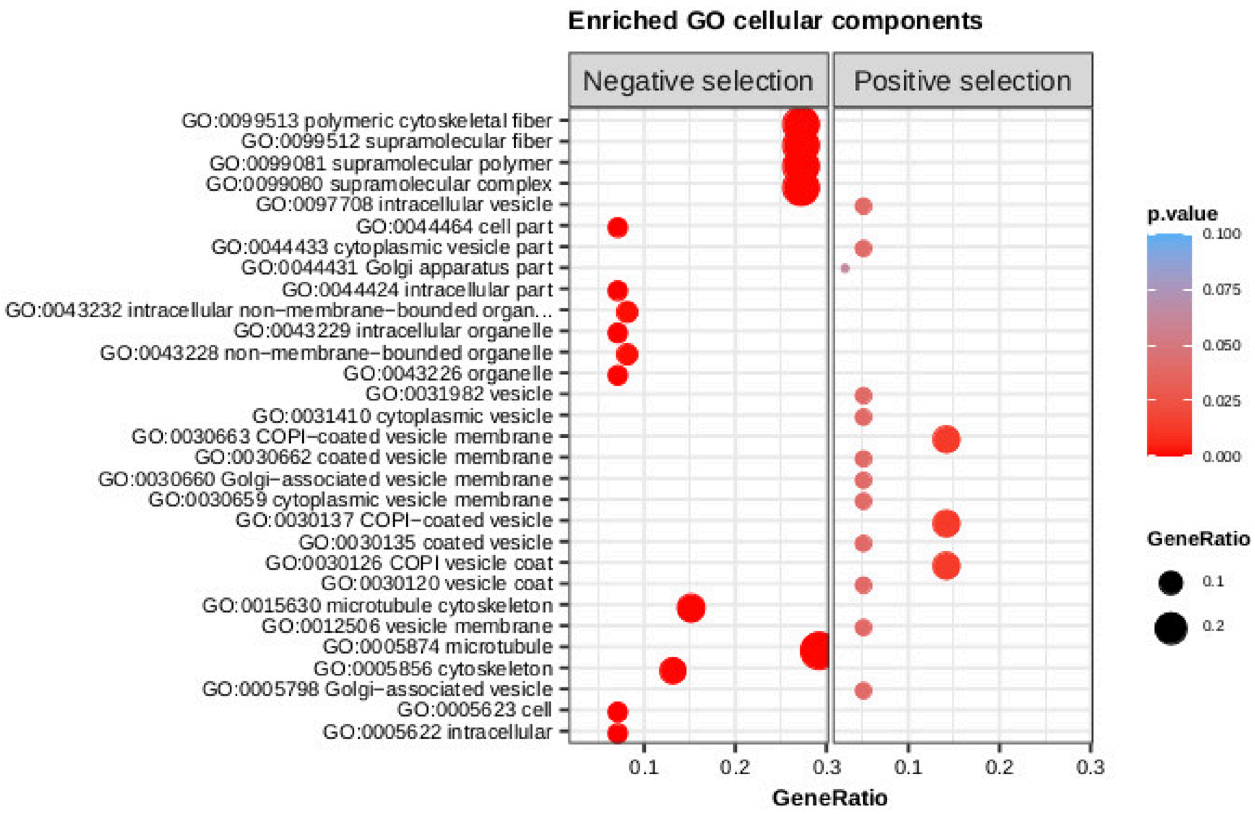

Supplementary Figure 8. Enrichment of genes under selection pressure for **A)** molecular function **B)** Cellular components.

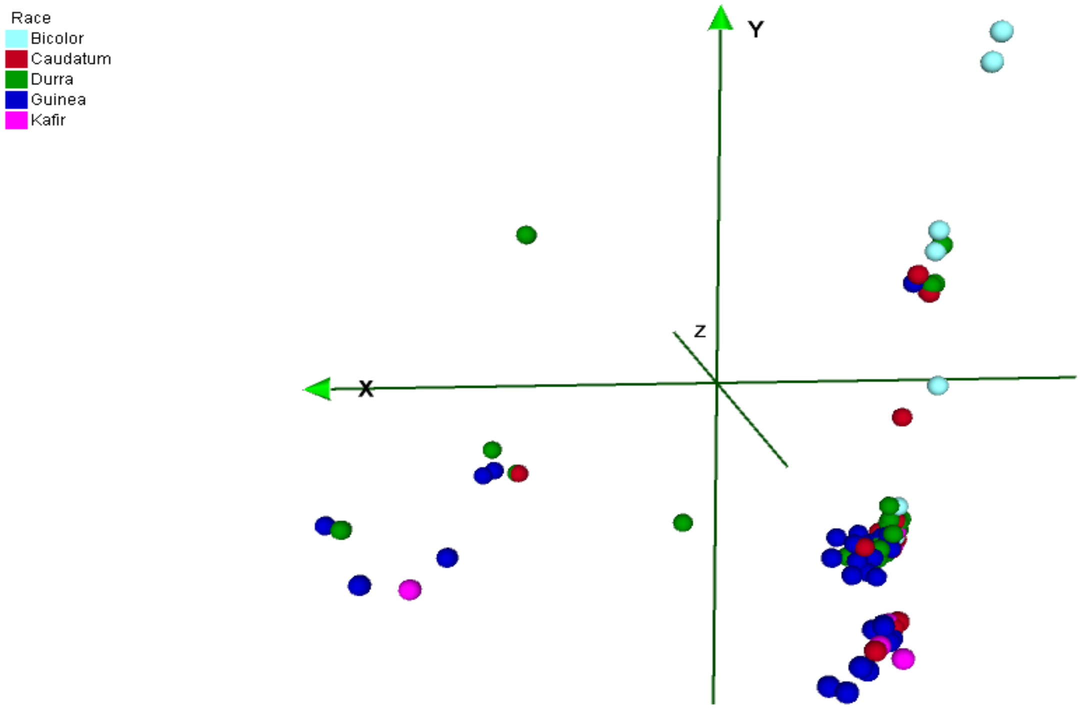

Supplementary Figure 9: PCO analysis of sorghum accessions shows the clusters of accessions with different races.

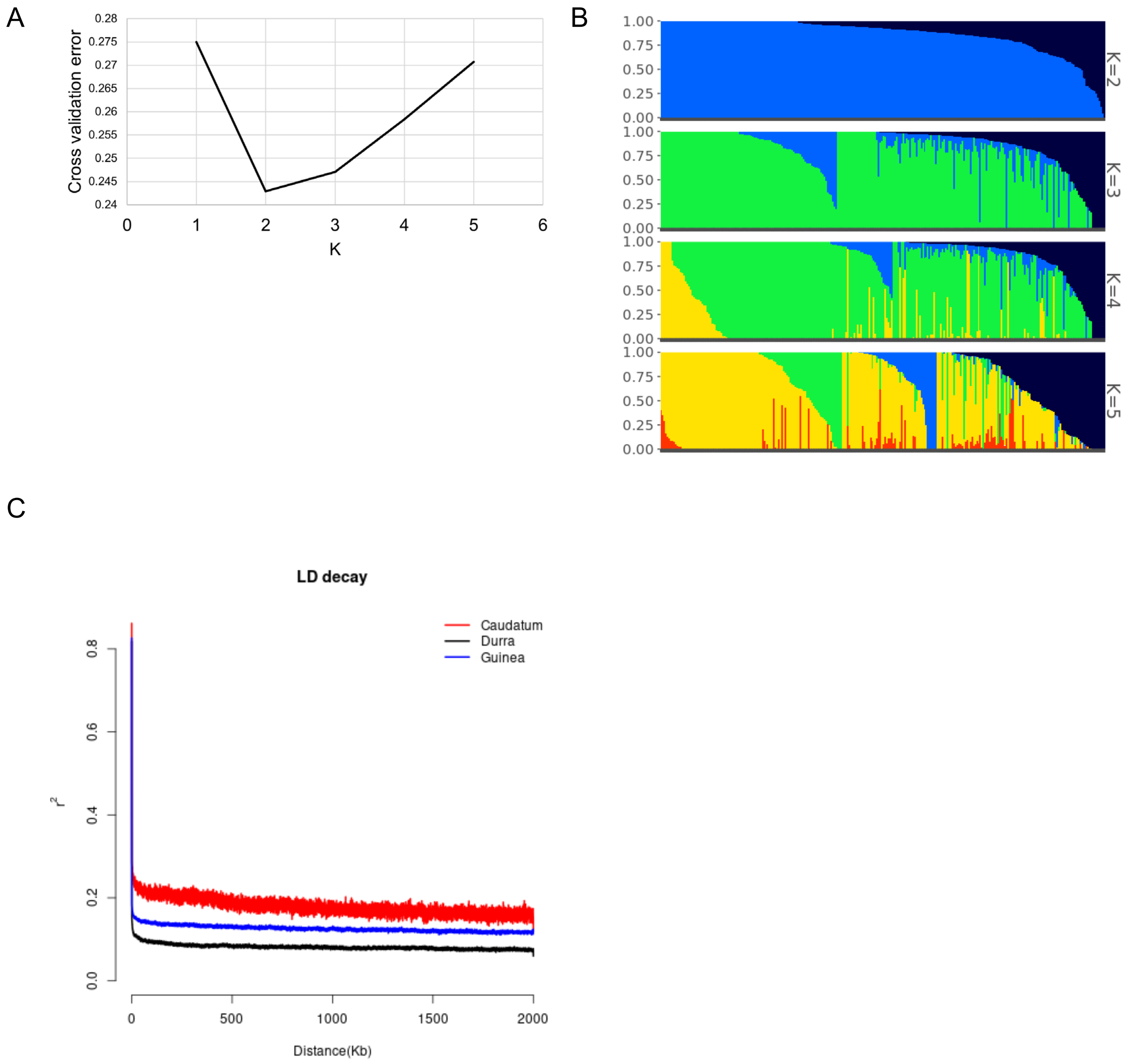

Supplementary Figure 10: Structure analysis of the sorghum race population shows A) the low cross validation error for K 2,3 B) the population structure with different K values and C) the LD decay in the sorghum race population having more than 50 accessions. Rapid LD decay was observed in Durra followed by Guinea and Caudatum.
